# Antiproliferative and Proapoptotic Effects of Phenanthrene Derivatives Isolated from *Bletilla striata* on A549 Lung Cancer Cells

**DOI:** 10.3390/molecules27113519

**Published:** 2022-05-30

**Authors:** Fei Zhou, Rui Feng, Ou Dai, Lian Yang, Yu Liu, Yun-Cai Tian, Cheng Peng, Liang Xiong

**Affiliations:** 1 State Key Laboratory of Southwestern Chinese Medicine Resources, School of Pharmacy, Chengdu University of Traditional Chinese Medicine, Chengdu 611137, China; zhoufeizhufeifff@163.com (F.Z.); fengrui4613@163.com (R.F.); 2 School of Pharmacy, Chengdu University of Traditional Chinese Medicine, Chengdu 611137, China; yanglian080910@163.com (L.Y.); liuyuyy519@163.com (Y.L.); 3 Institute of Innovative Medicine Ingredients of Southwest Specialty Medicinal Materials, School of Pharmacy, Chengdu University of Traditional Chinese Medicine, Chengdu 611137, China; 4 Shanghai Zhizhenzhichen Technologies Co., Ltd., Shanghai 201415, China; tianyuncai126@126.com

**Keywords:** *Bletilla striata*, phenanthrenes, A549 lung cancer cells, antiproliferation, proapoptosis

## Abstract

Lung cancer continues to be the world’s leading cause of cancer death and the treatment of non-small cell lung cancer (NSCLC) has attracted much attention. The tubers of *Bletilla striata* are regarded as “an excellent medicine for lung diseases” and as the first choice to treat several lung diseases. In this study, seventeen phenanthrene derivatives, including two new compounds (**1** and **2**), were isolated from the tubers of *B. striata*. Most compounds showed cytotoxicity against A549 cells. An EdU proliferation assay, a cell cycle assay, a wound healing assay, a transwell migration assay, a flow cytometry assay, and a western blot assay were performed to further investigate the effect of compound **1** on A549 cells. The results showed that compound **1** inhibited cell proliferation and migration and promoted cell apoptosis in A549 cells. The mechanisms might correlate with the regulation of the Akt, MEK/ERK, and Bcl-2/Bax signaling pathways. These results suggested that the phenanthrenes of *B. striata* might be important and effective substances in the treatment of NSCLC.

## 1. Introduction

For decades, lung cancer has been a considerable health issue owing to its high incidence and fatality rates [1,2]. Indeed, lung cancer kills more than one million people worldwide every year, with 80–90% of cases being related to NSCLC [3]. Although our understanding of targeted therapies, immunotherapy, and genetic alterations of cancers is evolving, the cure rate for NSCLC remains low [4,5]. In recent years, natural products from traditional Chinese medicines have been suggested as potential drugs to treat NSCLC [6,7]. Therefore, the discovery of anti-NSCLC active ingredients from traditional Chinese medicines has become one of the hotspots of modern lung cancer research.

**Figure 1:**
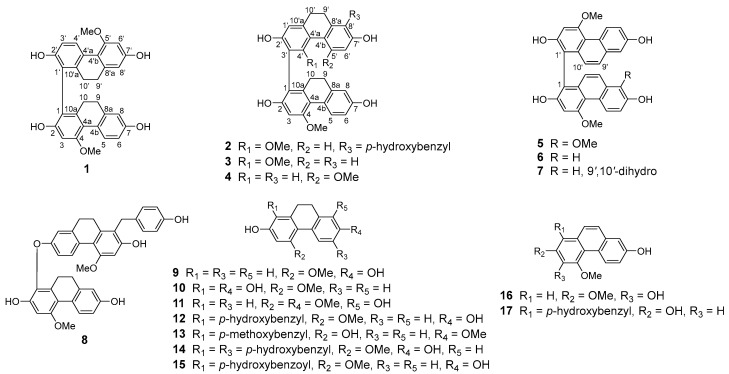
Structures of phenanthrenes **1**–**17** from *B. striata*.

The tubers of *Bletilla striata* (Thunb.) Rchb.f. (Orchidaceae), *Baiji* in Chinese, are a noted traditional Chinese medicine [8]. The effective use of tubers of *Bletilla striata* in the treatment of tuberculosis, silicosis, and hemoptysis caused by lung vessel damage has given *B. striata* the reputation of “an excellent medicine for lung diseases” [9,10]. Several papers have reported that phenanthrenes and bibenzyls isolated from *B. striata* exert significant cytotoxic activities against A549 cells, suggesting a potential therapeutic effect on NSCLC [11,12,13]. Our prior research has found that EtOAc extract and bibenzyls exhibited remarkable cytotoxic activity against A549 cells [14]. To further enrich our understanding of the functions of *B. striata* on lung cancer, phenanthrene derivatives and their cytotoxicity against A549 cells were investigated. Two new phenanthrene derivatives (**1** and **2**), together with 15 known analogues, blestrianol A (**3**) [15], gymconopin C (**4**) [16], bleformin G (**5**) [17], blestriarene C (**6**) [18], blestriarene B (**7**) [18], blestanol C (**8**) [13], 2,7-dihydroxy-4-methoxy-9,10-dihydrophenanthrene (**9**) [19], 4-methoxy-9,10-dihydrophenanthrene-1,2,7-triol (**10**) [20], 1,7-dihydroxy-2,5-dimethoxy-9,10-dihydrophenanthrene (**11**) [19], 1-(*p*-hydroxybenzyl)-4-methoxy-9,10-dihydrophenanthrene-2,7-diol (**12**) [21], pleioanthrenin (**13**) [22], 1,6-bis(*p*-hydroxybenzyl)-4-methoxy-9,10-dihydrophenanthrene-2,7-diol (**14**) [23], 2,7-dihydroxy-l-(*p*-hydroxybenzoyl)-4-methoxy-9,10-dihydrophenanthrene (**15**) [24], 3,7-dihydroxy-2,4-dimethoxyphenanthrene (**16**) [25], and 1-(*p*-hydroxybenzyl)-4-methoxyphenanthrene-2,7-diol (**17**) [23], were isolated from EtOAc extract from *B. striata* (Figure 1). Among them, 14 compounds showed significant cytotoxicity against A549 cells. Furthermore, the antiproliferative and proapoptotic effects of compound **1**, and their underlying mechanisms, were further explored.

## 2. Results

### 2.1. Structure Elucidation

Compound **1** showed IR absorption peaks at 1455 and 1589 cm^−1^ for aromatic rings and 3227 cm^−1^ for hydroxy groups. Its molecular formula was determined as C_30_H_26_O_6_ with 18 degrees of unsaturation from a (−)-HR-ESI-MS ion peak at 481.1647 [M − H]^−^ (calculated for C_30_H_25_O_6_, 481.1651). The ^13^C-NMR data of **1** (Table 1) displayed 30 signals attributed to four methylenes, two aromatic methoxys, and 24 aromatic carbons, which revealed the existence of four phenyls in **1**. Correspondingly, the ^1^H-NMR and ^1^H−^1^H COSY spectra indicated a pentasubstituted phenyl (δ_H_ 6.61, s), a 1,3,4-trisubstituted phenyl (δ_H_ 8.09 (d, *J* = 8.4 Hz), 6.69 (dd, *J* = 8.4, 2.4 Hz), and 6.66 (d, *J* = 2.4 Hz)), a 1,2,3,4-tetrasubstituted phenyl (δ_H_ 8.10 (d, *J* = 8.4 Hz) and 6.80 (d, *J* = 8.4 Hz)), a 1,3,4,5-tetrasubstituted phenyl (δ_H_ 6.47 (d, *J* = 2.4 Hz) and 6.35 (d, *J* = 2.4 Hz)), four phenolic hydroxys (δ_H_ 7.34, 7.43, 8.10, and 8.24), two aromatic methoxys (δ_H_ 3.87 and 3.89, each 3H, s), and two -CH_2_-CH_2_- fragments (δ_H_ 2.58, 2.50, 2.38, and 2.31, each 2H, m). These spectroscopic data indicate that compound **1** is a 9,9′,10,10′-tetrahydrobiphenanthrene derivative [17]. The connection between the two 9,10-dihydrophenanthrene monomers, as well as the locations of the hydroxy and methoxy groups, was determined by 2D NMR experiments. The HMBC correlations from H-3 to C-1, C-2, C-4, and C-4a; from H_2_-9 to C-4b, C-8, C-8a, and C-10a; from H_2_-10 to C-1, C-4a, C-8a, C-9, and C-10a; from H-3′ to C-1′ and C-4′a; from H_2_-9′ to C-4′b, C-8′, C-8′a, and C-10′a; and from H_2_-10′ to C-1′, C-4′a, C-8′a, C-9′, and C-10′a revealed the 1,1′-linkage of compound **1**. In addition, the HMBC correlations from OMe-4 to C-4; from OMe-5′ to C-5′; from OH-2 to C-1, C-2, and C-3; from OH-7 to C-6, C-7, and C-8; from OH-2′ to C-1′, C-2′, and C-3′; and from OH-7′ to C-6′, C-7′, and C-8′ verified that the two methoxy and four hydroxy groups are linked to C-4, C-5′, C-2, C-7, C-2′, and C-7′, respectively. Therefore, compound **1** was determined to be 4,5′-dimethoxy-9,9′,10,10′-tetrahydro-[1,1′-biphenanthrene]-2,2′,7,7′-tetraol.

**Figure 2:**
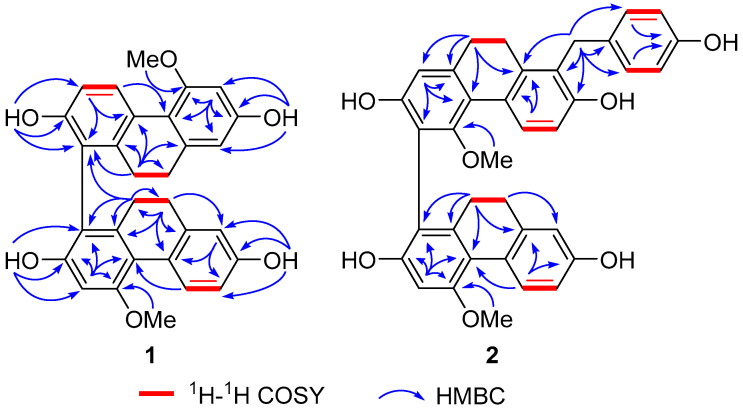
Key ^1^H–^1^H COSY and HMBC correlations of compounds **1** and **2**.

Compound **2** is also a 9,9′,10,10′-tetrahydrobiphenanthrene derivative, as indicated by its similar UV, IR, and NMR data to compound **1**. A quasimolecular ion peak at 587.2061 [M − H]^−^ in (−)-HR-ESI-MS determined the molecular formula of **2** as C_37_H_32_O_7_ possessing 22 degrees of unsaturation, which suggested an additional benzyl group in **2**. A comparison of the ^1^H- and ^13^C-NMR data between compounds **2** and **3** (blestrianol A) [17] revealed that an additional *p*-hydroxybenzyl group (δ_H_ 7.08 (2H, d, *J* = 9.0 Hz), 6.71 (2H, d, *J* = 9.0 Hz), 4.11 (1H, d, *J* = 15.6 Hz), and 4.03 (1H, d, *J* = 15.6 Hz); δ_C_ 115.8 (2 × C), 130.0 (2 × C), 132.9, 156.1, and 31.5) was substituted on the biphenanthrene moiety in compound **2**. Thus, compound **2** is *p*-hydroxybenzyl-substituted blestrianol A. This conjecture was verified by 2D NMR data analysis. In particular, the HMBC correlations of H-7″ with C-7′, C-8′, and C-8a′ proved that the *p*-hydroxybenzyl was linked to C-8′. Furthermore, the HMBC correlations from H-3 to C-1, C-2, C-4, and C-4a; from H_2_-10 to C-1, C-4a, C-8a, and C-10a; from H-1′ to C-3′, C-4′a, and C-10′; from H_2_-10′ to C-1′, C-4′a, C-8′a, and C-10′a; from OMe-4 to C-4; and from OMe-4′ to C-4′, together with the ^1^H–^1^H COSY correlations of H-5/H-6 and H-5′/H-6′, confirmed the 1,3′-connection between the two dihydrophenanthrene monomers and the positions of four hydroxy and two methoxy groups. Therefore, compound **2** was determined to be 4,4′-dimethoxy-8′-(*p*-hydroxybenzyl)-9,9′,10,10′-tetrahydro-[1,3′-biphenanthrene]-2,2′,7,7′-tetraol. Key ^1^H–^1^H COSY and HMBC correlations of compounds **1** and **2** were shown in Figure 2.

### 2.2. Cytotoxicity of the Isolates against A549 and BEAS-2B Cells

Our prior research showed that EtOAc extract from *B. striata* exhibited significant cytotoxic activity against A549 cells (IC_50_ = 11.92 ± 0.68 μg/mL). However, water extract and *n*-BuOH extract from *B. striata* exhibited no obvious cytotoxicity (IC_50_ > 100 μg/mL) [14]. Thus, the cytotoxic effects of the phenanthrenes isolated from the EtOAc extract were investigated in this study. Table 2 presents the cytotoxicity results of the isolates against A549 lung cancer cells; compounds **11** and **15** were not detected due to limited sample amounts after structure determination. In addition, the cytotoxicity of compound **1** against normal human lung cells (BEAS-2B) was also assessed. The result showed that the cytotoxic effect of compound **1** on BEAS-2B cells (IC_50_ = 15.22 ± 1.62 μM) was much weaker than that on A549 cells (IC_50_ = 6.86 ± 0.71 μM).

### 2.3. Compound ***1*** Inhibited Cell Proliferation and Induced Cell Cycle Arrest at G2/M Phase of A549 Cells

**Figure 3:**
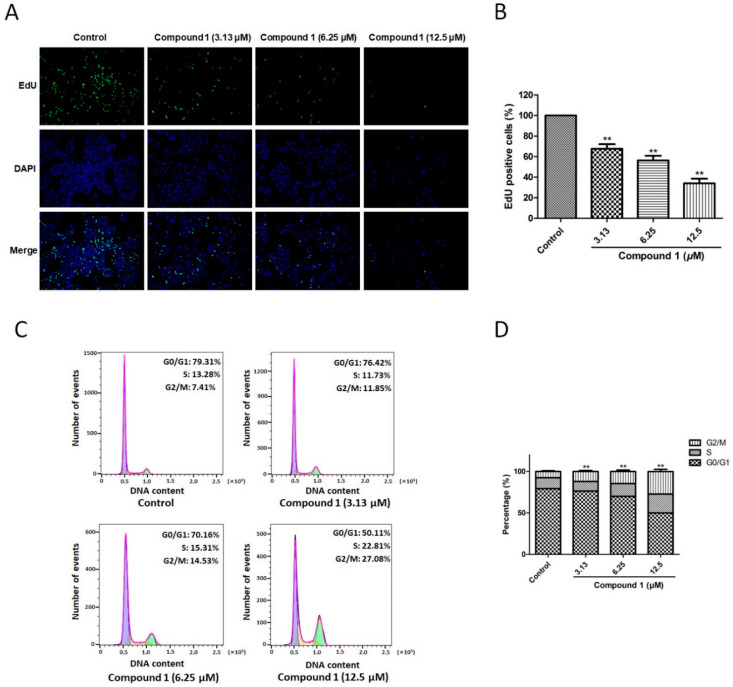
Effect of compound **1** on proliferation of A549 cells. (**A**) Images of control, compound **1** (3.13 µM), compound **1** (6.25 µM), and compound **1** (12.5 µM) groups were captured by a microscope in an EdU staining assay. (**B**) Treatment with 3.13, 6.25, and 12.5 µM of compound **1** notably reduced EdU-positive cells. (**C**) Cell cycle analysis of control, compound **1** (3.13 µM), compound **1** (6.25 µM), and compound **1** (12.5 µM) groups were performed by flow cytometry. (**D**) Treatment with 3.13, 6.25, and 12.5 µM of compound **1** dramatically increased the percentage of cells in the G2/M phase. Data are shown as the mean ± SD (*n* = 3). ** *p* < 0.01 in comparison with the control group.

To measure the proliferative effect of compound **1** on A549 cells, EdU immunofluorescence labeling was used. As shown in Figure 3A,B, after treatment with compound **1** (3.13, 6.25, and 12.5 μM), the percentage of EdU-positive cells decreased from 100% to 67.66 ± 4.51% (*p* < 0.01), 56.33 ± 4.50% (*p* < 0.01), and 34.01 ± 4.58% (*p* < 0.01), respectively. Furthermore, cell cycle analysis was carried to investigate the antiproliferative effect of compound **1** on A549 cells. Figure 3C,D indicated that the percentage of G2/M phase cells in the untreated control was 7.41 ± 0.79%, while compound **1** at 3.13, 6.25, and 12.5 μM increased this percentage to 11.85 ± 1.26% (*p* < 0.01), 14.53 ± 1.75% (*p* < 0.01), and 27.08 ± 2.65% (*p* < 0.01), respectively. All the data indicated that compound **1** induced cell cycle arrest at G2/M phase, causing the proliferation inhibition of A549 cells.

### 2.4. Compound ***1*** Inhibited Migration of A549 Cells

**Figure 4:**
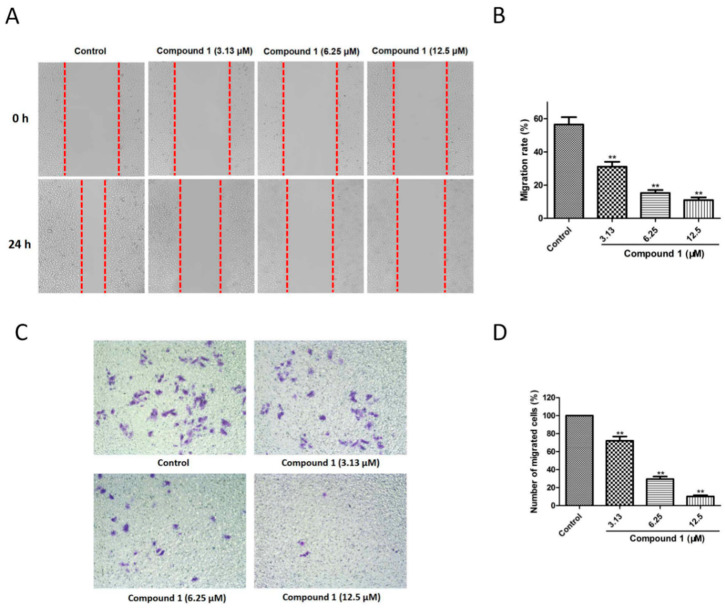
Effect of compound **1** on migration of A549 cells. (**A**) Microscopy images obtained during wound healing assay of control, compound **1** (3.13 µM), compound **1** (6.25 µM), and compound **1** (12.5 µM) groups. (**B**) Treatment with 3.13, 6.25, and 12.5 µM of compound **1** markedly decreased the migration rate. (**C**) Microscopy images acquired during transwell migration assay of control, compound **1** (3.13 µM), compound **1** (6.25 µM), and compound **1** (12.5 µM) groups. (**D**) Treatment with 3.13, 6.25, and 12.5 µM of compound **1** dramatically reduced the migrated cell numbers. Data are shown as the mean ± SD (*n* = 3). ** *p* < 0.01 in comparison with the control group.

The migratory effect of compound **1** on A549 cells was investigated by migration-related assays. As shown in Figure 4A, at concentrations of 3.13, 6.25, and 12.5 µM, compound **1** increased the scratch area in a dose-dependent manner. The migration rate decreased from 56.46 ± 4.46% in the control group to 31.10 ± 2.92% (*p* < 0.01), 15.23 ± 1.81% (*p* < 0.01), and 11.01 ± 1.81% (*p* < 0.01) at 3.13, 6.25, and 12.5 µM of compound **1**, respectively (Figure 4B). Meanwhile, the transwell migration assay showed that, at concentrations of 3.13, 6.25, and 12.5 µM, compound **1** significantly reduced the migrated cell numbers from 100% to 72.09 ± 4.65% (*p* < 0.01), 29.45 ± 2.92% (*p* < 0.01), and 10.19 ± 1.25% (*p* < 0.01), respectively (Figure 4C,D). Thus, compound **1** notably suppressed the migration of A549 cells in a dose-dependent manner.

### 2.5. Compound ***1*** Induced Apoptosis of A549 Cells

**Figure 5:**
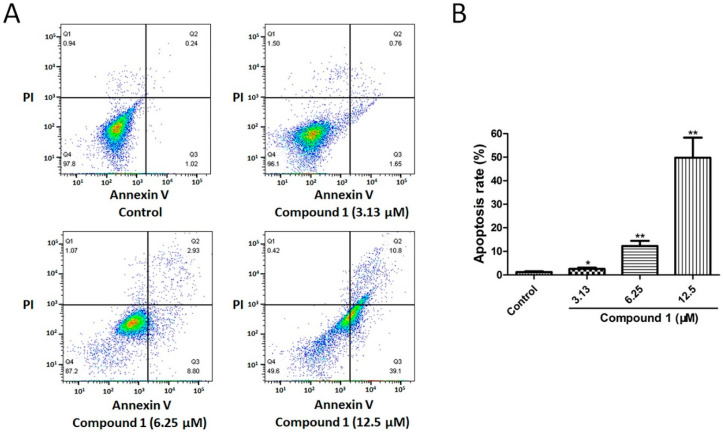
Effect of compound **1** on apoptosis of A549 cells. (**A**) Cell apoptosis in control, compound **1** (3.13 µM), compound **1** (6.25 µM), and compound **1** (12.5 µM) groups was detected via flow cytometry. (**B**) Treatment with 3.13, 6.25, and 12.5 µM of compound **1** induced apoptosis of A549 cells. Data are shown as the mean ± SD (*n* = 3). * *p* < 0.05 and ** *p* < 0.01 in comparison with the control group.

Quadrants 2 (Annexin V+/PI+) and 3 (Annexin V+/PI−) pointed to the percentage of apoptotic cells. As for the control group, the apoptosis rate was only 1.21 ± 0.37%. After a 48-h treatment with 3.13, 6.25, and 12.5 µM of compound **1**, the apoptosis rates remarkably increased to 2.57 ± 0.53% (*p* < 0.05), 12.25 ± 2.21% (*p* < 0.01), and 49.79 ± 8.54% (*p* < 0.01), respectively (Figure 5). The data disclosed that compound **1** greatly promoted the apoptosis of A549 cells, especially in the high-concentration group (12.5 µM).

### 2.6. Compound ***1*** Inhibited Proliferation of A549 Cells via Suppressing the Akt and MEK/ERK Signaling Pathways

**Figure 6:**
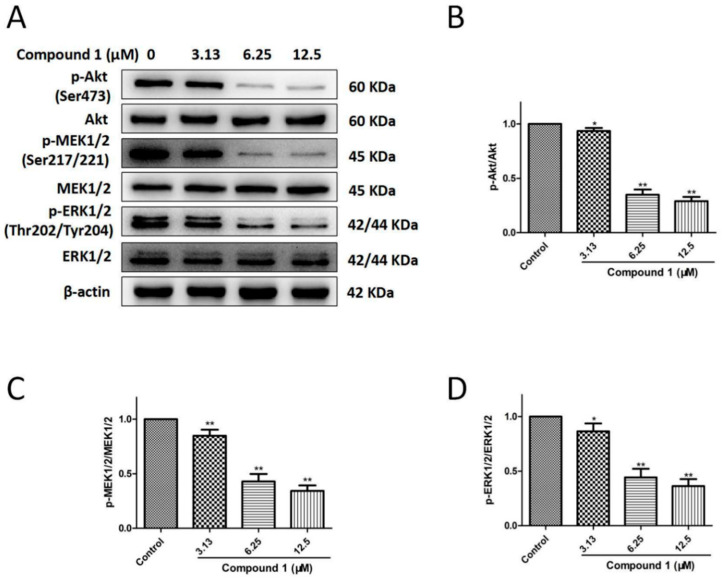
Effects of compound **1** on the Akt and MEK/ERK signaling pathways in A549 cells. (**A**) The phosphorylation and total protein levels of Akt, MEK1/2, and ERK1/2 were detected with an immunoblotting assay. (**B**) Compound **1** at concentrations of 3.13, 6.25, and 12.5 μM notably decreased the phosphorylation level of Akt. (**C**) Compound **1** at concentrations of 3.13, 6.25, and 12.5 μM obviously reduced the phosphorylation level of MEK1/2. (**D**) Compound **1** at concentrations of 3.13, 6.25, and 12.5 μM significantly reduced the phosphorylation level of ERK1/2. Data are shown as the mean ± SD (*n* = 3). * *p* < 0.05 and ** *p* < 0.01 in comparison with the control group.

The antiproliferative mechanisms of compound **1** were further explored via western blotting (Figure 6 and Table S1). Compound **1** (3.13, 6.25, and 12.5 µM) significantly suppressed the expression ratios of p-Akt/Akt (*p* < 0.05 at 3.13 µM, *p* < 0.01 at 6.25 and 12.5 μM), p-MEK/MEK (*p* < 0.01 at 3.13, 6.25, and 12.5 μM), and p-ERK/ERK (*p* < 0.05 at 3.13 μM, *p* < 0.01 at 6.25 and 12.5 μM) in comparison with the control group. Therefore, compound **1** inhibited the proliferation of A549 cells by inhibiting Akt and MEK/ERK phosphorylation.

### 2.7. Compound ***1*** Induced Apoptosis of A549 Cells by Regulating Bcl-2/Bax Signaling Pathway

**Figure 7:**
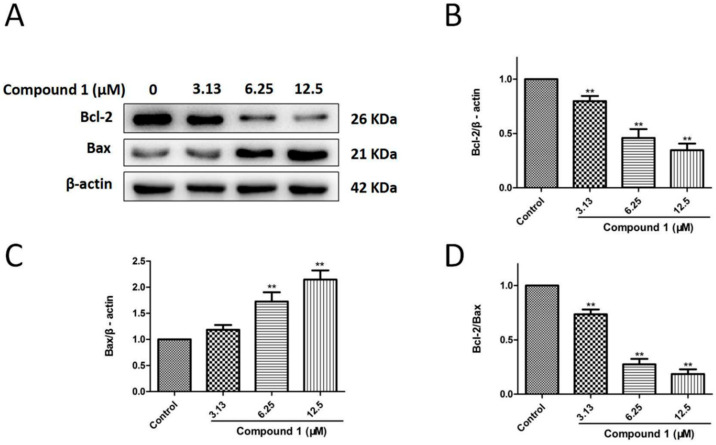
Effects of compound **1** on Bcl-2 and Bax expression in A549 cells. (**A**) Bcl-2 and Bax protein levels were detected by an immunoblotting experiment. (**B**) Compound **1** at concentrations of 3.13, 6.25, and 12.5 μM significantly decreased the expression level of Bcl-2. (**C**) Compound **1** at concentrations of 6.25 and 12.5 μM significantly increased the expression level of Bax. (**D**) Compound **1** at concentrations of 3.13, 6.25, and 12.5 μM dramatically reduced the Bcl-2/Bax ratio. Data are shown as the mean ± SD (*n* = 3). ** *p* < 0.01 in comparison with the control group.

Figure 7 and Table S2 suggested that compound **1** significantly decreased Bcl-2 protein levels (*p* < 0.01 at 3.13, 6.25, and 12.5 μM), increased Bax protein levels (*p* < 0.01 at 6.25 and 12.5 μM), and reduced the Bcl-2/Bax ratio (*p* < 0.01 at 3.13, 6.25, and 12.5 μM) in a dose-dependent manner versus the control group. Apparently, compound **1** promoted apoptosis of A549 cells by downregulating the Bcl-2/Bax expression ratio.

## 3. Discussion

Considering its high incidence and mortality, lung cancer is gradually becoming a serious health problem [26,27]. NSCLC is the most frequent type of lung cancer according to the histological type [28]. Although surgery, radiation, and immunotherapy have been used for treating NSCLC, pharmaceutical treatment is of great significance, and innovative drugs are needed. Traditional Chinese medicine (TCM) reflects the profound understanding of the Chinese people regarding life, health, and disease and has a time-honored historical tradition and unique theories and techniques. With the advantages of diverse chemical structures, a wide range of sources, and significant activities, natural products from TCM have been studied for the treatment of NSCLC [29,30]. Therefore, it is meaningful and feasible to find effective natural compounds from TCM to treat NSCLC.

The tubers of *B. striata*, praised as “an effective medicine to treat lung diseases”, were the best choice in terms of lung disease treatment according to Shennong’s Materia Medica, Essential of Materia Medica and Treasury of Words on Materia Medica. Modern studies have demonstrated that phenanthrene derivatives from *B. striata* exert significant cytotoxic activities against A549 cells [11,13,31,32]. Therefore, this study explored phenanthrene derivatives from EtOAc extract from *B. striata* in terms of cytotoxicity against A549 cells and investigated the preliminary mechanisms. As expected, 17 phenanthrene derivatives, including two new compounds (**1** and **2**), were isolated from the tubers of *B. striata*. Most of the tested compounds showed cytotoxicity, especially compounds **1**, **2**, **4**, **6**, **7**, **8**, and **13** (IC_50_ < 10 μM). Moreover, the structure–cytotoxicity relationship was also investigated. In general, the cytotoxic effects of biphenanthrenes (**1**–**4** and **6**–**8**) were much stronger than those of simple phenanthrenes (**9**, **10**, **12**, **14**, **16**, and **17**). However, the IC_50_ value of compound **5** was over 100 μM. A comparison of the results for compound **5** and other biphenanthrenes, especially between compounds **5** and **6**, indicated that the introduction of an OMe group in position eight resulted in a considerable reduction in cytotoxicity. In addition, a comparison of compounds **1** (1,1′-connection) and **4** (1,3′-connection) showed that the manner of the connection between the two dihydrophenanthrene monomers did not significantly affect the cytotoxicity. In the simple phenanthrenes, when compared with compound **9**, the introduction of an additional OH (**10**) or an additional *p*-hydroxybenzyl (**12**) at C-1 significantly improved the cytotoxicity. The introduction of another *p*-hydroxybenzyl at C-6 (**14**) further notably increased the cytotoxicity. These structure–activity relationships may serve as a reference in the investigation of anti-NSCLC phenanthrenes from *B. striata*.

The novel compound **1**, possessing good cytotoxicity, was used to study the preliminary mechanisms. EdU immunofluorescence staining and cell cycle analysis were carried out to detect the antiproliferation effect of compound **1** on A549 cells. The data showed that, at concentrations of 3.13, 6.25, and 12.5 µM, compound **1** significantly inhibited the proliferation of A549 cells and induced cell cycle arrest at the G2/M phase. The checkpoint at the G2/M transition is a crucial regulatory gate during cell-cycle progression, but the cell will die if the cell-cycle checkpoint is lost before the completion of DNA repair [33,34]. In addition, cell migration is an essential procedure when the metastatic dissemination of cancer cells is mentioned [35]. Compound **1** exhibited an inhibitory effect with respect to A549 cell migration, as determined by the migration-related assays. Since Akt dominates the growth, cycle, metabolism, and death of cells by regulating various downstream substrates [36], the effect of compound **1** on AKT phosphorylation in A549 cells was investigated. Meanwhile, MAPKs acts as one of the significant serine/threonine protein kinases, which exhibit a crucial role in receiving signals and transmitting them into cytomembrane [37]. ERK1/2 is identified as an important mitogen-activated factor, which participates in numerous biological processes including both cell proliferation and survival. MEK1/2 is a crucial upstream protein of ERK1/2 [38]. Both of them are important members of the MAPK family, and the MEK/ERK signaling pathway has been proven to be crucial for NSCLC research [39,40]. Thus, the expression ratios of p-MEK/MEK and p-ERK/ERK were evaluated. These results disclosed that the Akt and MEK/ERK signaling pathways were involved in the antiproliferation effect of compound **1** on A549 cells.

Unlike necrosis, apoptosis is a type of programmed cell death. Apoptosis disorder can cause pathological events. Among them, tumors and autoimmune diseases are representative examples [41]. The flow cytometry results indicated that compound **1** dramatically promoted the apoptosis of A549 cells. Thus, the proapoptotic effect of compound **1** was further studied. Bcl-2 and Bax are regarded as important apoptotic regulatory proteins. Bcl-2 promotes cell survival and suppresses cell death, while the effects of Bax are the opposite [42,43]. Our results pointed out that the proapoptotic effect of compound **1** on A549 cells was associated with a decrease in the Bcl-2/Bax ratio.

## 4. Materials and Methods

### 4.1. General Procedures

A Bruker AVIIIHD-600 spectrometer was used to collect NMR data (Bruker Corporation, Billerica, MA, USA). HR-ESI-MS analyses were carried out on a Synapt G2 HDMS instrument (Waters Corporation, Milford, MA, USA). IR data was obtained using an Agilent Cary 600 FT-IR microscope (Agilent Technologies Inc., Santa Clara, CA, USA). Silica gel (200–300 mesh; Yantai Institute of Chemical Technology, Yantai, China), polyamide sorbent (30–60 mesh; Shanghai Yien Chemical Technology Co., Ltd., Shanghai, China), MCI gel CHP 20P (75–150 μm, Mitsubishi Chemical, Co., Tokyo, Japan), and Sephadex LH-20 (40–70 μm, Amersham Pharmacia Biotech AB, Uppsala, Sweden) were used for column chromatography. An instrument equipped with an Ultimate (250 × 10 mm^2^) semi-preparative column (C18, 5 μm), a Cometro 6000PVW UV/VIS detector, and a Cometro 6000LDS pump was used for HPLC separations. Glass precoated silica gel GF254 plates were used for TLC (Qingdao Marine Chemical Inc., Qingdao, China). Gibco provided the RPMI-1640 medium (Grand Island, NY, USA). Hyclone supplied the FBS (South Logan, UT, USA). MTT was purchased from Sigma-Aldrich (St. Louis, MO, USA). Beyotime Institute of Biotechnology (Shanghai, China) provided EdU and DAPI. BD Biosciences (San José, CA, USA) provided the matrigel. Nanjing KeyGen Biotech. Co. Ltd. (Nanjing, China) supplied the Cell Cycle Detection Kit and Annexin V-FITC/PI Apoptosis Detection Kit. Cell Signaling Technology (CST; Danvers, MA, USA) provided rabbit anti-phospho-Akt (Ser473), rabbit anti-Akt, rabbit anti-ERK1/2 antibodies, rabbit anti-phospho-ERK1/2 (Thr202/Tyr204), and rabbit anti-phospho-MEK1/2 (Ser217/221). Chengdu Zen Bioscience Co., Ltd. (Chengdu, China) provided the rabbit anti-MEK1/2 antibody. Abcam (Cambridge, UK) provided rabbit anti-Bcl-2 and rabbit anti-Bax antibodies. GeneTex, Inc. (Irvine, CA, USA) provided the rabbit anti-β-actin antibody. Zsbio Commerce Store (Beijing, China) supplied peroxidase-conjugated AffiniPure goat anti-rabbit immunoglobulin G (IgG; [H + L]; ZB-2301). A549 cells were made available by the American Tissue Culture Collection (ATCC, Rockville, MD, USA), and BEAS-2B cells were purchased from Guangzhou Huatuo Biological Technology Co., Ltd. (Guangzhou, China). All analytical solvents were obtained from Chengdu Kelong Chemical Reagent Factory (Chengdu, China).

### 4.2. Plant Material

The tubers of *B. striata* (voucher specimen: SBS-121023) were gathered in October 2012 in Neijiang, Sichuan, China. Professor Min Li provided help in the identification of medicinal materials. The sample was stored in the State Key Laboratory of Southwestern Chinese Medicine Resources in Sichuan, China.

### 4.3. Extraction and Isolation

The tubers of *B. striata* (3 kg) were refluxed three times with 95% EtOH for 2 h each time. The decompressing of the concentration of EtOH extract yielded a semi-solid residue (510 g). The residue was dispersed in H_2_O and extracted with petroleum ether, EtOAc, and *n*-BuOH in turn. After removal of the solvent, the EtOAc fraction (160 g) was put to silica gel column chromatography with a gradient elution of increasing Me_2_CO (0–100%) in petroleum ether to yield 25 major fractions (Fr. 1–Fr. 25). Separation of fraction Fr. 16 (25 g) gave 10 fractions (Fr. 16-1–Fr. 16-10) using an MCI column (35%, 50%, 65%, 80%, and 95% MeOH in H_2_O). Fraction Fr. 16-5 was separated via silica gel column chromatography to yield seven sections (Fr. 16-5a–Fr. 16-5g). Compound **9** (300 mg) was crystallized from Fr. 16-5a in MeOH. Successive purification of Fr. 16-5c by preparative TLC (CH_2_Cl_2_/MeOH, 15:1) and HPLC (68% MeOH in H_2_O) yielded compounds **1** (13.5 mg), **3** (13.2 mg), **4** (20.2 mg), and **10** (12.8 mg). Fraction Fr. 16-6 was fractioned by a silica gel column (CH_2_Cl_2_/MeOH, 1:0–3:2) to yield four subfractions (Fr. 16-6a–Fr. 16-6d). Fr. 16-6b was purified by Sephadex LH-20 chromatography column (CH_2_Cl_2_/MeOH, 1:1), preparative TLC (CH_2_Cl_2_/MeOH, 30:1), and semipreparative HPLC (60% MeOH in H_2_O) to yield compounds **11** (0.5 mg) and **16** (7.8 mg). RP-MPLC (20–100% MeOH in H_2_O) was used to separate Fr. 16-7 into seven subfractions (Fr. 16-7a–Fr. 16-7g). Compounds **12** (11.2 mg) and **15** (0.6 mg) were obtained via purifying Fr. 16-7c by a silica gel column (CH_2_Cl_2_/MeOH, 100:0–4:1), a Sephadex LH-20 column (65% MeOH in H_2_O), and preparative TLC (CH_2_Cl_2_/MeOH, 20:1). Fraction Fr. 16-10 was separated by Sephadex LH-20 column chromatography eluted with CH_2_Cl_2_/MeOH (1:1), followed by repeated preparative TLC (CH_2_Cl_2_/MeOH, 20:1) and semipreparative HPLC (55% MeCN in H_2_O) to yield compound **13** (9.8 mg).

Fraction Fr. 18 (19 g) was divided into 17 subfractions (Fr. 18-1–Fr. 18-17) via a polyamide column using a gradient elution of EtOH/H_2_O (20:80–95:5). Sephadex LH-20 column chromatography (CH_2_Cl_2_/MeOH, 1:1) was used to divide Fr. 18-13 into six subfrations (Fr. 18-13a–Fr. 18-13f). Fr. 18-13b was purified by a Sephadex LH-20 column (70% MeOH in H_2_O), followed by preparative TLC (CH_2_Cl_2_/MeOH, 10:1), and semipreparative HPLC (60% MeOH in H_2_O) to yield compound **2** (3.8 mg). Compounds **14** (8.5 mg) and **17** (5.2 mg) were obtained from Fr. 18-13c by Sephadex LH-20 column chromatography (CH_2_Cl_2_/MeOH, 1:1) and repeated preparative TLC (CH_2_Cl_2_/MeOH, 15:1). Compounds **7** (8.6 mg) and **5** (12.8 mg) were isolated from Fr. 18-13d and Fr. 18-13e, respectively, by Sephadex LH-20 column chromatography (CH_2_Cl_2_/MeOH, 1:1) and repeated preparative TLC (CH_2_Cl_2_/MeOH, 10:1). Similarly, purification of Fr. 18-13f yielded compounds **6** (23.9 mg) and **8** (7.8 mg).

4,5′-Dimethoxy-9,9′,10,10′-tetrahydro-[1,1′-biphenanthrene]-2,2′,7,7′-tetraol (**1**): yellowish powder; UV (MeCN) *λ*_max_ (log *ε*) 214 (4.32), 281 (4.05) nm; IR *ν*_max_ 3227, 2931, 2842, 1589, 1455, 1344, 1202,1159,1078, 992, 944, 825, 714, 627 cm^−1^; ^1^H-NMR (600 MHz, acetone-*d*_6_) and ^13^C-NMR (150 MHz, acetone-*d*_6_) data, see Table 1; (−)-HR-ESI-MS *m*/*z* 481.1647 [M − H]^−^ (calculated for C_30_H_25_O_6_, 481.1651).

4,4′-Dimethoxy-8′-(*p*-hydroxybenzyl)-9,9′,10,10′-tetrahydro-[1,3′-biphenanthrene]-2,2′,7,7′-tetraol (**2**): yellowish powder; UV (MeCN) *λ*_max_ 213 (4.38), 276 (4.15) nm; IR *ν*_max_ 3384, 2922, 2852, 1641, 1364, 1202, 1033, 921, 764, 724, 628 cm^−1^; ^1^H-NMR (600 MHz, acetone-*d*_6_) and ^13^C-NMR (150 MHz, acetone-*d*_6_) data, see Table 1; (−)-HR-ESI-MS *m*/*z* 587.2061 [M − H]^−^ (calculated for C_37_H_31_O_7_, 587.2070).

### 4.4. Cytotoxic Activity Assay

The purity of the tested compounds was more than 98%. The cytotoxic effects of isolated phenanthrenes on A549 and BEAS-2B cells were examined by MTT experiments as described in our previous report [14].

### 4.5. EdU Proliferation Experiment

The cells were digested by trypsin and cultivated in 96-well plates (3.5 × 10^3^ cells per well) for 24 h. They were incubated with compound **1** (3.13, 6.25, and 12.5 μM) for 48 h. Next, prepared EdU-labeling solution (10 µM) was used to stain the cells in an incubator at 37 °C for 2 h. The cells were fixed for 15 min with 4% cold paraformaldehyde. After that, Triton X-100 (0.3%) and Click-iT reaction cocktail were successively used to handle cells. Finally, DAPI staining was applied to the cells to counterstain them. A Leica DMI3000B inverted fluorescence microscope (Leica, Wetzlar, Germany) was used to capture the fluorescent images. In addition, to count the cells, ImageJ software (version 1.8.0) (National Institutes of Health, Bethesda, MD, USA) was used. EdU positive cells (%) = (green EdU-stained cells/blue DAPI-stained cells) × 100.

### 4.6. Cell Cycle Analysis

According to the manufacture’s instructions, the cells were treated with compound **1** (3.13, 6.25, and 12.5 μM) for 48 h. Then, they were cultured with 500 μL staining solution (RNase A:PI = 1:9) for 1 h at room temperature without light after fixation with 70% pre-chilled ethanol for 2 h. The DNA content was instantly detected with a BD FACSCanto II flow cytometer (BD Biosciences, Franklin Lakes, NJ, USA).

### 4.7. Wound Healing Assay

Cells were placed in 6-well plates for the wound healing test. The scratches were formed using a sterile 200-µL pipette tip when the cells treated with compound **1** (3.13, 6.25, and 12.5 μM) had grown to 90% confluence. After a 24-h incubation, the images were obtained with the Leica microscope and the scratch area was calculated with the ImageJ. Migration rate (%) = [(A0 − A1)/A0] × 100, where A0 represents scratch area at 0 h, and A1 suggests scratch area at 24 h.

### 4.8. Transwell Migration Assay

The migratory effect of compound **1** (3.13, 6.25, and 12.5 μM) on A549 cells was further detected by using transwell chambers. Briefly, the cells treated with compound **1** were placed into the upper wells, while the medium with 10% FBS was added to the bottom wells. After a 24-h incubation, non-migrated cells were cleaned up, and the migrated cells were fixed for 30 min with 4% paraformaldehyde and dyed by 0.1% crystal violet. The images and the cell numbers were obtained by the Leica microscope and ImageJ, respectively.

### 4.9. Apoptosis by Flow Cytometry Assay

The A549 cells were treated with compound **1** (3.13, 6.25, and 12.5 μM), and the cell apoptosis was detected by flow cytometry assay as described in our previous report [14].

### 4.10. Western Blot Analysis

After a 48-h incubation with compound **1** (3.13, 6.25, and 12.5 μM), the proteins of the cells were extracted using RIPA buffer with proteinase inhibitors. SDS–PAGE and PVDF membranes were used to separate and transfer the protein, respectively. Then, the membranes had a 2-h incubation with 5% nonfat milk at room temperature and an overnight incubation with primary antibodies at 4 °C [anti-β-actin (1:1000), anti-p-Akt (1:1000), anti-Akt (1:1000), anti-p-MEK1/2 (1:1000), anti-MEK1/2 (1:1000), anti-p-ERK1/2 (1:1000), anti-ERK1/2 (1:1000), anti-Bcl-2 (1:1000), and anti-Bax (1:1000)]. The membranes were washed with TBST and incubated with an HRP-conjugated secondary antibody for 1 h at 37 °C. Finally, the protein bands were detected with a Tanon 5200 chemiluminescent imaging system (Tanon, Shanghai, China).

### 4.11. Statistical Analysis

Data are shown as mean ± standard deviation (SD) with three biological replicates. One-way ANOVA and Tukey’s post-hoc test were used to evaluate the significant differences (*p* < 0.05). Figures were obtained by GraphPad Prism software Version 5.0 (GraphPad Software, Inc., San Diego, CA, USA).

## 5. Conclusions

In this research, two new (**1** and **2**) and 15 known phenanthrene derivatives (**3**–**17**) were isolated from *B. striata*. Most compounds showed cytotoxicity against A549 cells. In particular, the IC_50_ values of compounds **1**, **2**, **4**, **6**, **7**, **8**, and **13** were less than 10 μM. The structure–cytotoxicity relationship indicated that the cytotoxic effects of biphenanthrenes are related to the polymerization degree and substituents (OMe, OH, and *p*-hydroxybenzyl groups). Compound **1** was further proven to have antiproliferative and proapoptotic effects of A549 cells. The mechanisms involved the regulation of the Akt, MEK/ERK, and Bcl-2/Bax signaling pathways.

## Figures and Tables

**Table 1:** ^1^H- (600 MHz) and ^13^C- (150 MHz) NMR data of compounds **1** and **2** (δ in ppm, *J* in Hz). NMR data were measured in acetone-*d*6.

No.	1		2	
	^1^H	^13^C	^1^H	^13^C
1		114.6		112.0
2		155.3		155.9
3	6.61 (s)	99.0	6.65 s	99.1
4		158.2		158.5
4a		116.9		117.1
4b		126.1		125.9
5	8.09 d (8.4)	130.1	8.09 d (8.4)	130.1
6	6.69 dd (8.4, 2.4)	113.3	6.69 dd (8.4, 2.4)	114.1
7		156.1		156.5
8	6.66 d (2.4)	114.7	6.69 d (2.4)	114.7
8a		140.2		140.3
9	2.47–2.53 m, 2.56–2.61 m	30.6	2.55–2.58 m, 2.59–2.64 m	30.3
10	2.27–2.34 m, 2.35–2.41 m	27.9	2.44–2.50 m, 2.51–2.55 m	28.3
10a		140.7		141.6
1′		121.5	6.69 s	114.7
2′		154.3		156.1
3′	6.80 d (8.4)	113.4		116.6
4′	8.10 d (8.4)	129.5		155.9
4′a		126.2		126.0
4′b		116.8		120.9
5′		158.7	8.10 d (8.4)	129.4
6′	6.47 d (2.4)	98.9	6.71 d (8.4)	113.4
7′		157.3		153.2
8′	6.35 d (2.4)	107.9		121.7
8′a		141.5		140.0
9′	2.47–2.53 m, 2.56–2.61 m	31.2	2.59–2.64 m, 2.66–2.70 m	27.1
10′	2.27–2.34 m 2.35–2.41 m	27.2	2.59–2.64 m, 2.66–2.70 m	30.3
10′a		139.7		140.0
1″				132.9
2″			7.08 d (9.0)	130.0
3″			6.71 d (9.0)	115.8
4″				156.1
5″			6.71 d (9.0)	115.8
6″			7.08 d (9.0)	130.0
7″			4.03 d (15.6), 4.11 d (15.6)	31.5
OMe-4	3.89 s	55.7	3.90 s	55.6
OMe-8				
OMe-4′			3.26 s	59.8
OMe-5′	3.87 s	55.7		
OH-2	7.43 s			
OH-7	8.10 s			
OH-2′	7.34 s			
OH-7′	8.24 s			

**Table 2:** Cytotoxicity of phenanthrenes **1**–**17** against A549 cells.

Compounds	IC_50_ (μM) ^a^	Compounds	IC_50_ (μM) ^a^
**1**	6.86 ± 0.71	**10**	16.29 ± 2.22
**2**	8.75 ± 0.61	**11**	- ^b^
**3**	10.50 ± 2.55	**12**	33.30 ± 0.49
**4**	9.91 ± 0.68	**13**	9.52 ± 0.47
**5**	>100	**14**	12.03 ± 0.79
**6**	9.45 ± 0.52	**15**	- ^b^
**7**	3.43 ± 0.21	**16**	63.80 ± 4.67
**8**	8.62 ± 0.64	**17**	32.61 ± 2.33
**9**	51.47 ± 1.87	Paclitaxel	(1.52 ± 0.02) × 10^−^^2^

^a^ Data are presented as mean ± SD, *n* = 3. ^b^ Cytotoxicity was not measured.

## Data Availability

The data presented in this study are available in the Supplementary Materials or can be provided by the authors.

## Supplementary Materials

The following supporting information can be downloaded at: https://www.mdpi.com/article/10.3390/molecules27113519/s1, Figures S1–S16 are the 1D and 2D NMR, HR-ESI-MS, IR, and UV spectra for two new compounds, Table S1: the original western blots in three repetitions for Figure 6 in the paper, Table S2: the original western blots in three repetitions for Figure 7 in the paper.

## Sample Availability

Samples of the compounds are available from the authors.
