# Supplementary material for: Antiproliferative and Proapoptotic Effects of Phenanthrene Derivatives Isolated from Bletilla striata on A549 Lung Cancer Cells
Source: Molecules. 2022 May 30;27(11):3519. doi: 10.3390/molecules27113519 (PMC9181924; doi:10.3390/molecules27113519)
Supplement: Supplementary file 1 [file molecules-27-03519-s001.zip › molecules-1692124-supplementary.pdf]

Supplementary material

Antiproliferative and proapoptotic effects of phenanthrene derivatives isolated from *Bletilla striata* on A549 lung cancer cells

The List of Contents

No	Content	Page
1	**Figure S1.** The UV spectrum of compound **1** in MeCN	S1
2	**Figure S2.** The IR spectrum of compound **1**	S2
3	**Figure S3.** The (–)-HR-ESI-MS spectroscopic data of compound **1**	S3
4	**Figure S4.** The ^{1}H NMR spectrum of compound **1** in acetone-d_6	S4
5	**Figure S5.** The ^{13}C NMR spectrum of compound **1** in acetone-d_6	S5
6	**Figure S6.** The HSQC spectrum of compound **1** in acetone-d_6	S6
7	**Figure S7.** The ^{1}H-^{1}H COSY spectrum of compound **1** in acetone-d_6	S7
8	**Figure S8.** The HMBC spectrum of compound **1** in acetone-d_6	S8
9	**Figure S9.** The UV spectrum of compound **2** in MeCN	S9
10	**Figure S10.** The IR spectrum of compound **2**	S10
11	**Figure S11.** The (–)-HR-ESI-MS spectroscopic data of compound **2**	S11
12	**Figure S12.** The ^{1}H NMR spectrum of compound **2** in acetone-d_6	S12
13	**Figure S13.** The ^{13}C NMR spectrum of compound **2** in acetone-d_6	S13
14	**Figure S14.** The HSQC spectrum of compound **2** in acetone-d_6	S14
15	**Figure S15.** The ^{1}H-^{1}H COSY spectrum of compound **2** in acetone-d_6	S15
16	**Figure S16.** The HMBC spectrum of compound **2** in acetone-d_6	S16
17	**Table S1.** The original western blots in three repetitions for Figure 6 in the paper	S17
18	**Table S2.** The original western blots in three repetitions for Figure 7 in the paper	S19

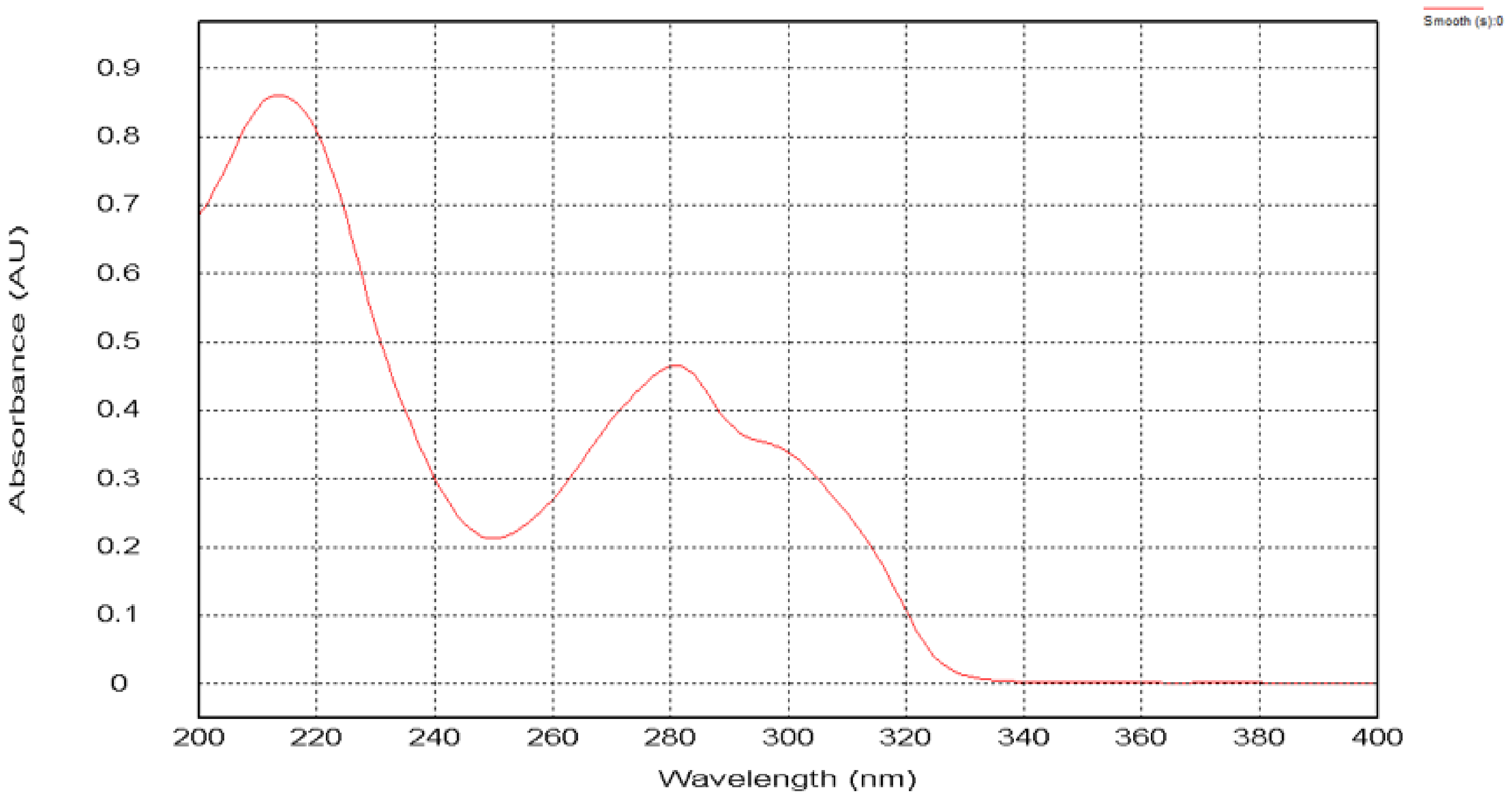

Figure S1. The UV spectrum of compound **1** in MeCN.

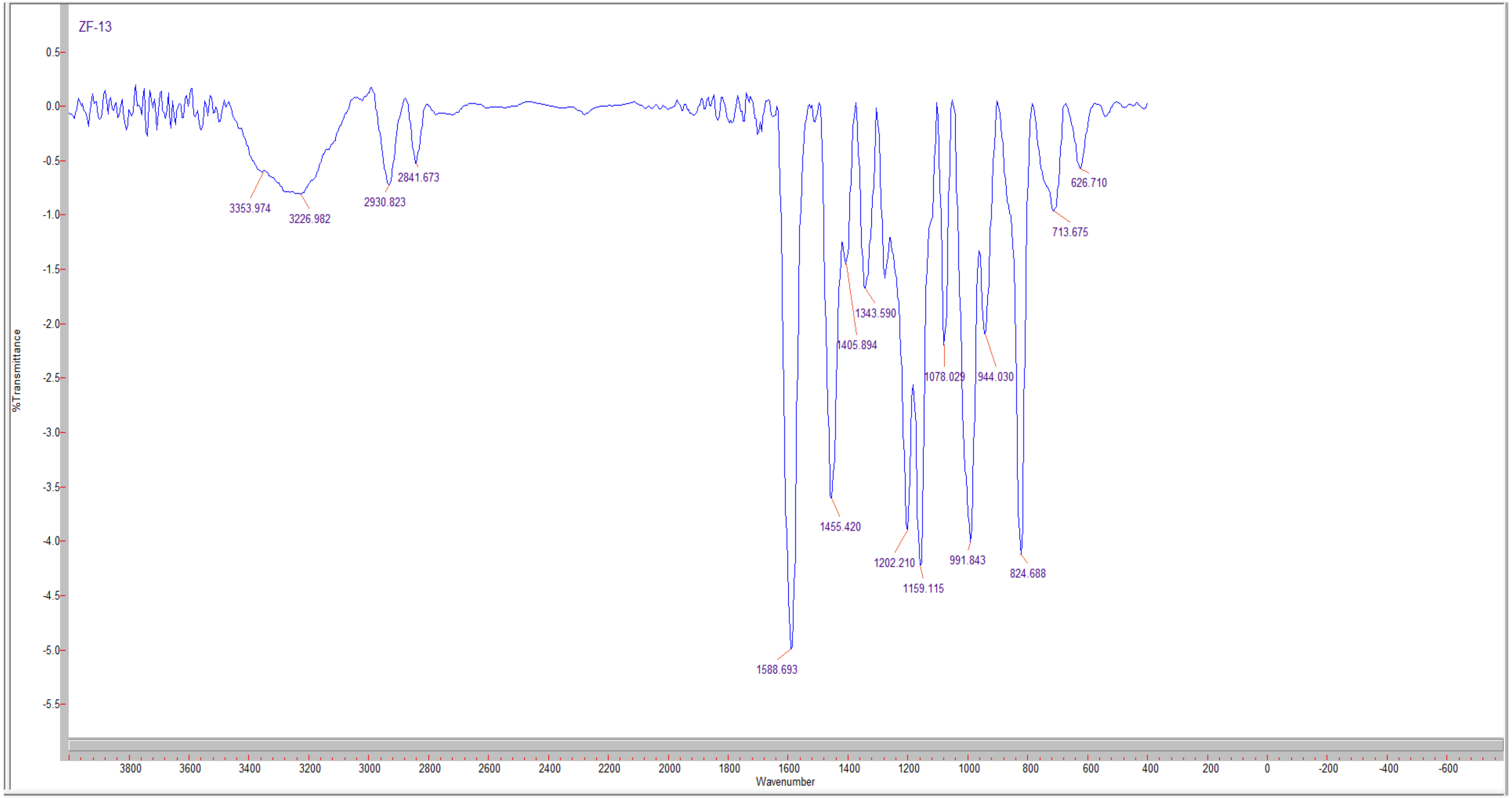

Figure S2. The IR spectrum of compound **1**.

Single Mass Analysis
Tolerance = 3.0 mDa / DBE: min = -1.5, max = 50.0
Element prediction: Off
Number of isotope peaks used for i-FIT = 3
Monoisotopic Mass, Even Electron Ions
100 formula(e) evaluated with 1 results within limits (up to 100 closest results for each mass)
Elements Used:

Mass	Calc. Mass	mDa	PPM	DBE	Formula	i-FIT	i-FIT Norm	Fit Conf %	C	H	O
481.1647	481.1651	-0.4	-0.8	18.5	C30 H25 O6	905.3	n/a	n/a	30	25	6

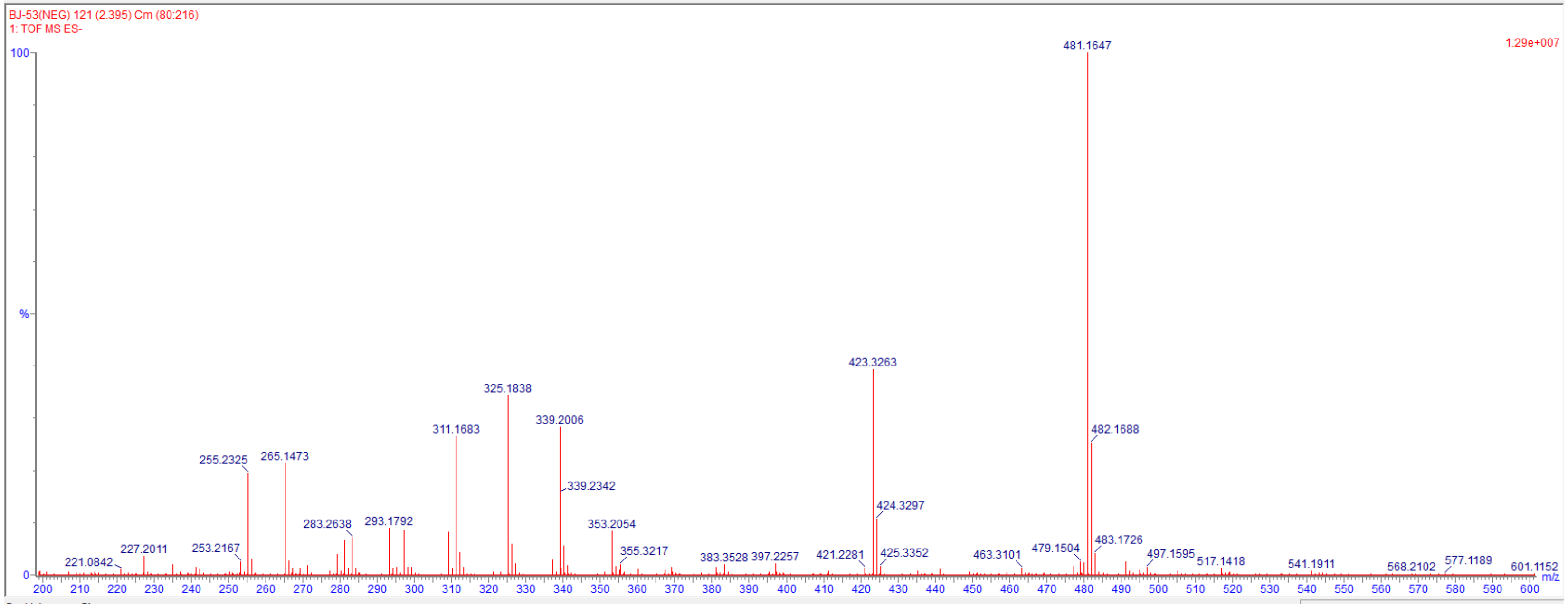

Figure S3. The (–)-HR-ESI-MS spectroscopic data of compound **1**.

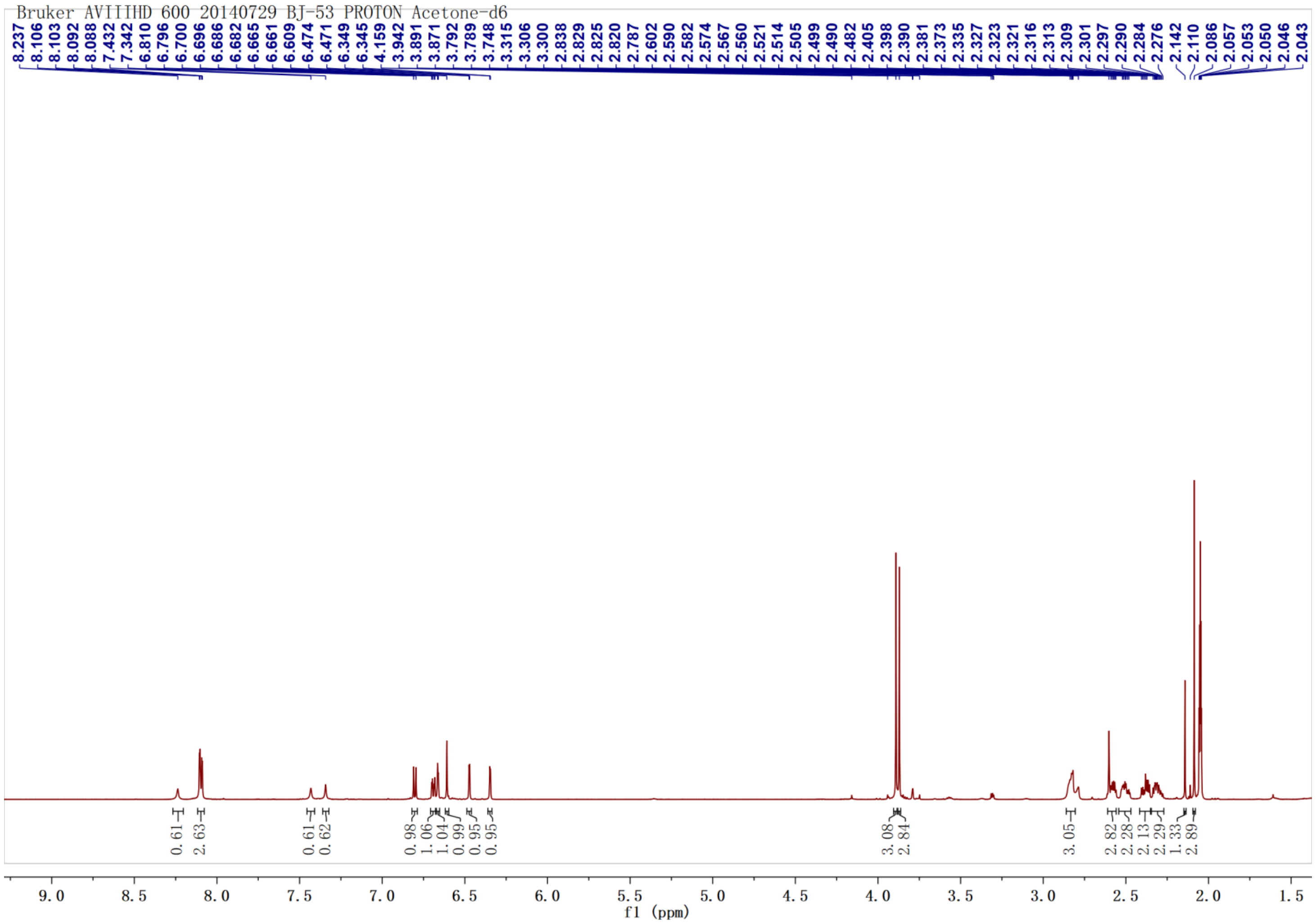

Figure S4. The ¹H NMR spectrum of compound **1** in acetone-d_6.

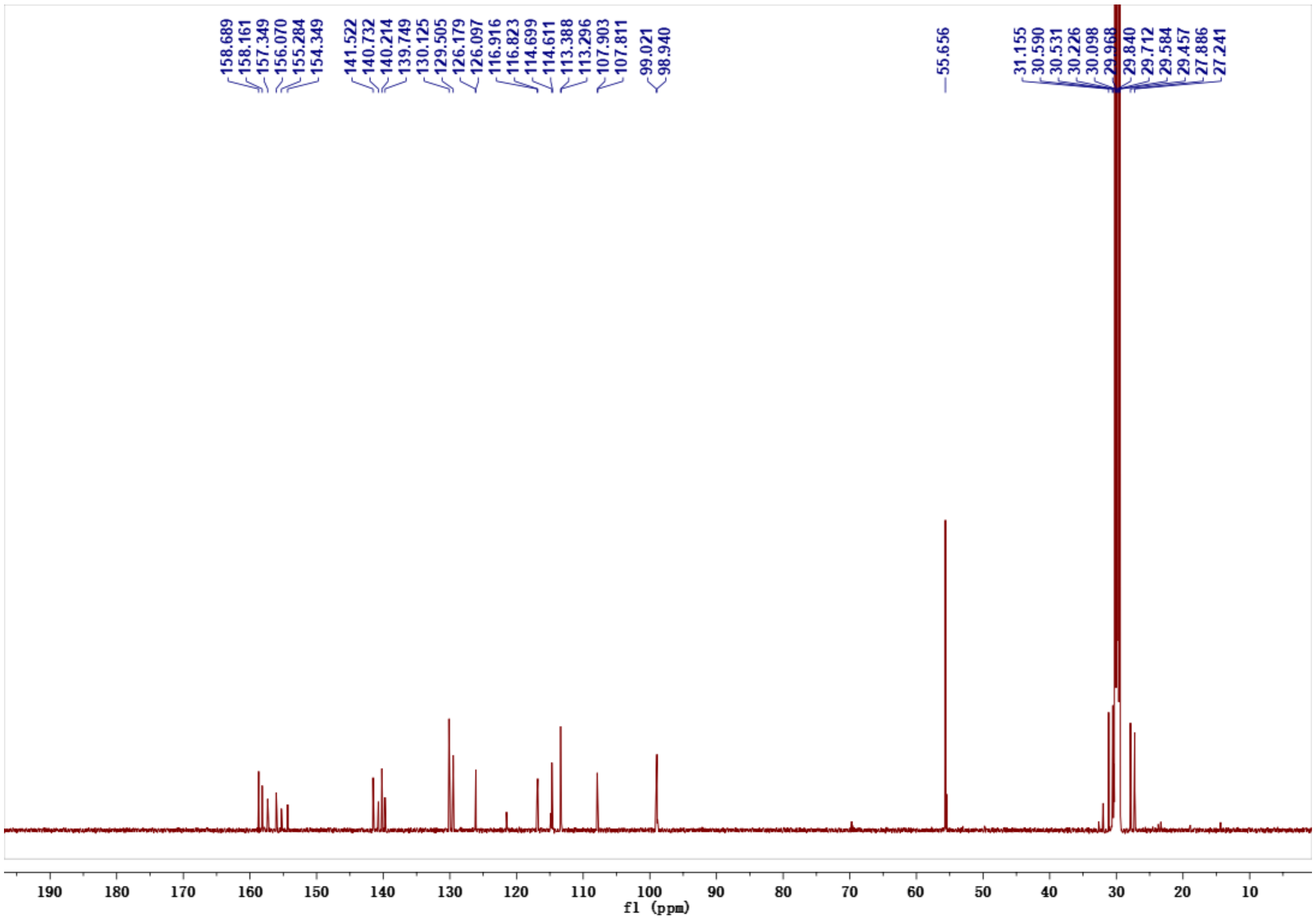

Figure S5. The ^{13}C NMR spectrum of compound **1** in acetone-d_6.

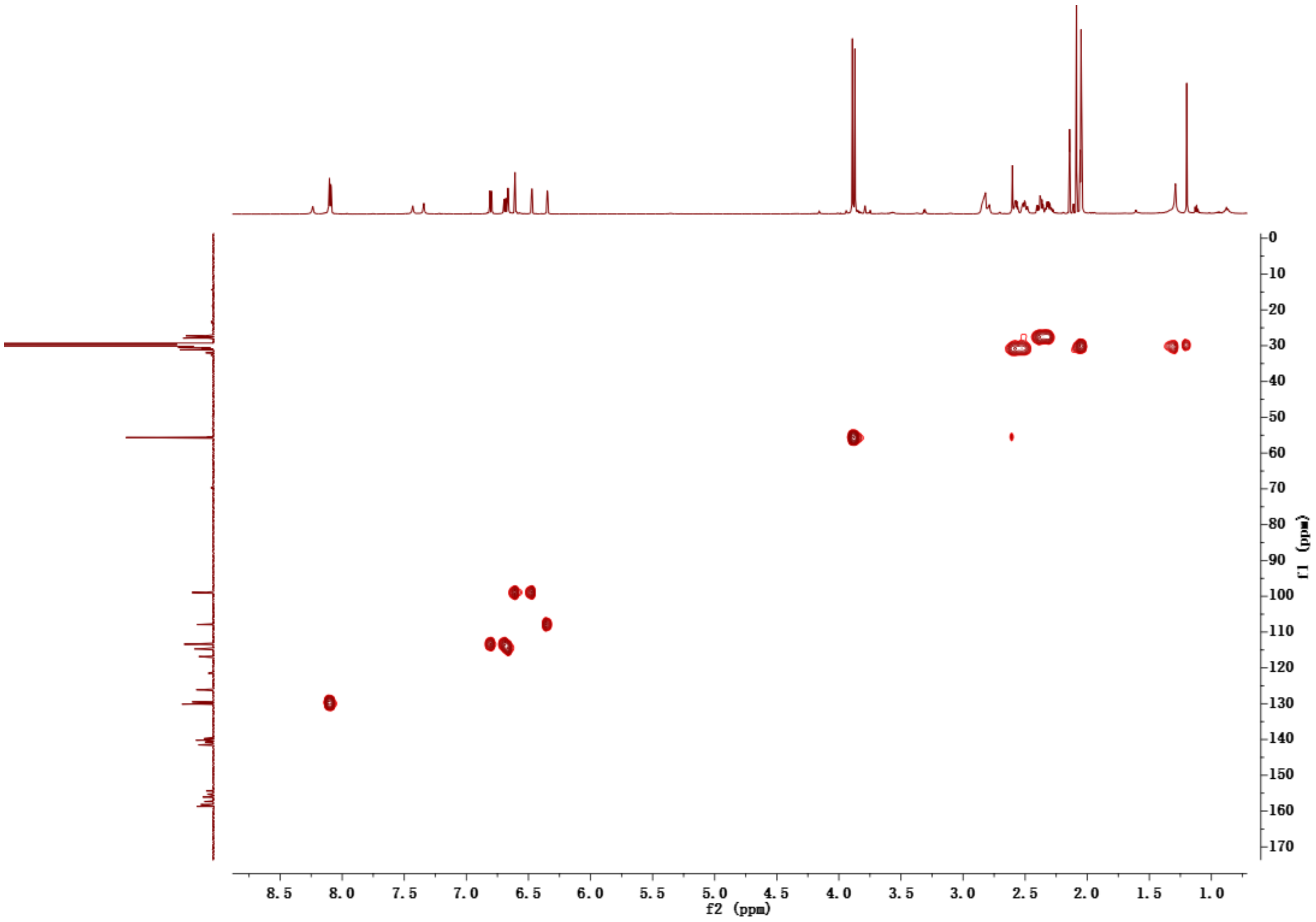

Figure S6. The HSQC spectrum of compound **1** in acetone-d_6.

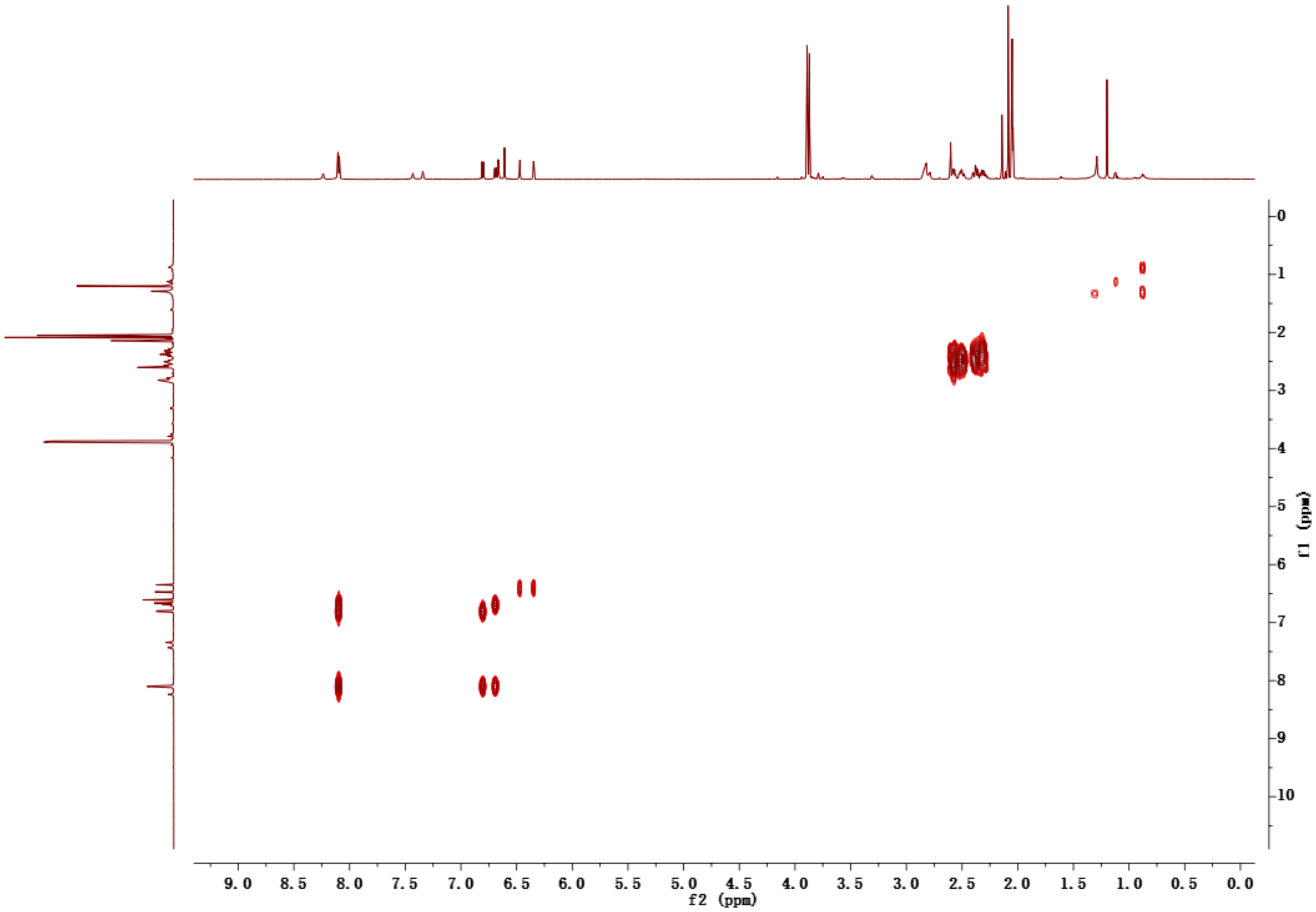

Figure S7. The ^{1}H-^{1}H COSY spectrum of compound **1** in acetone-d_6.

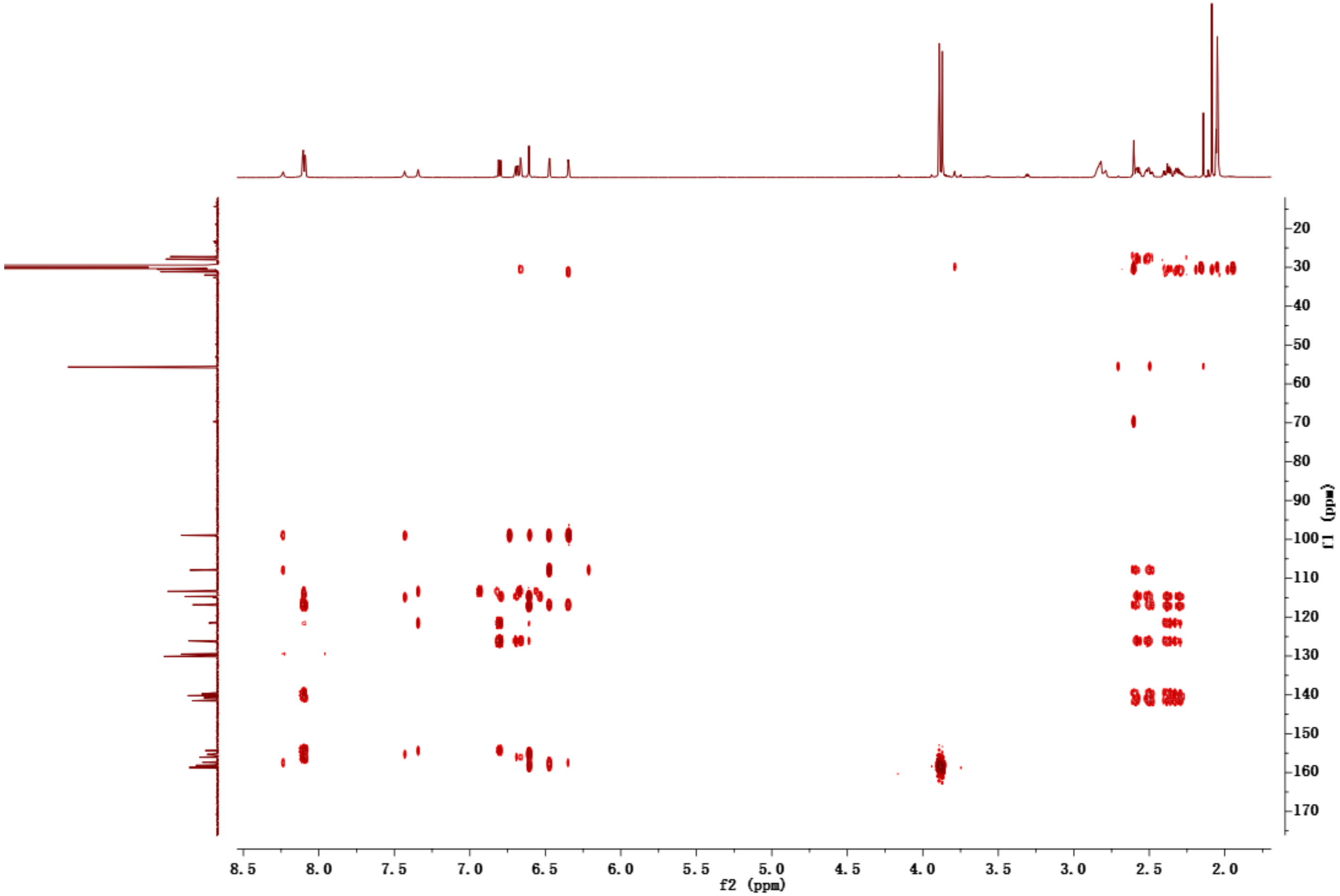

Figure S8. The HMBC spectrum of compound **1** in acetone-d_6.

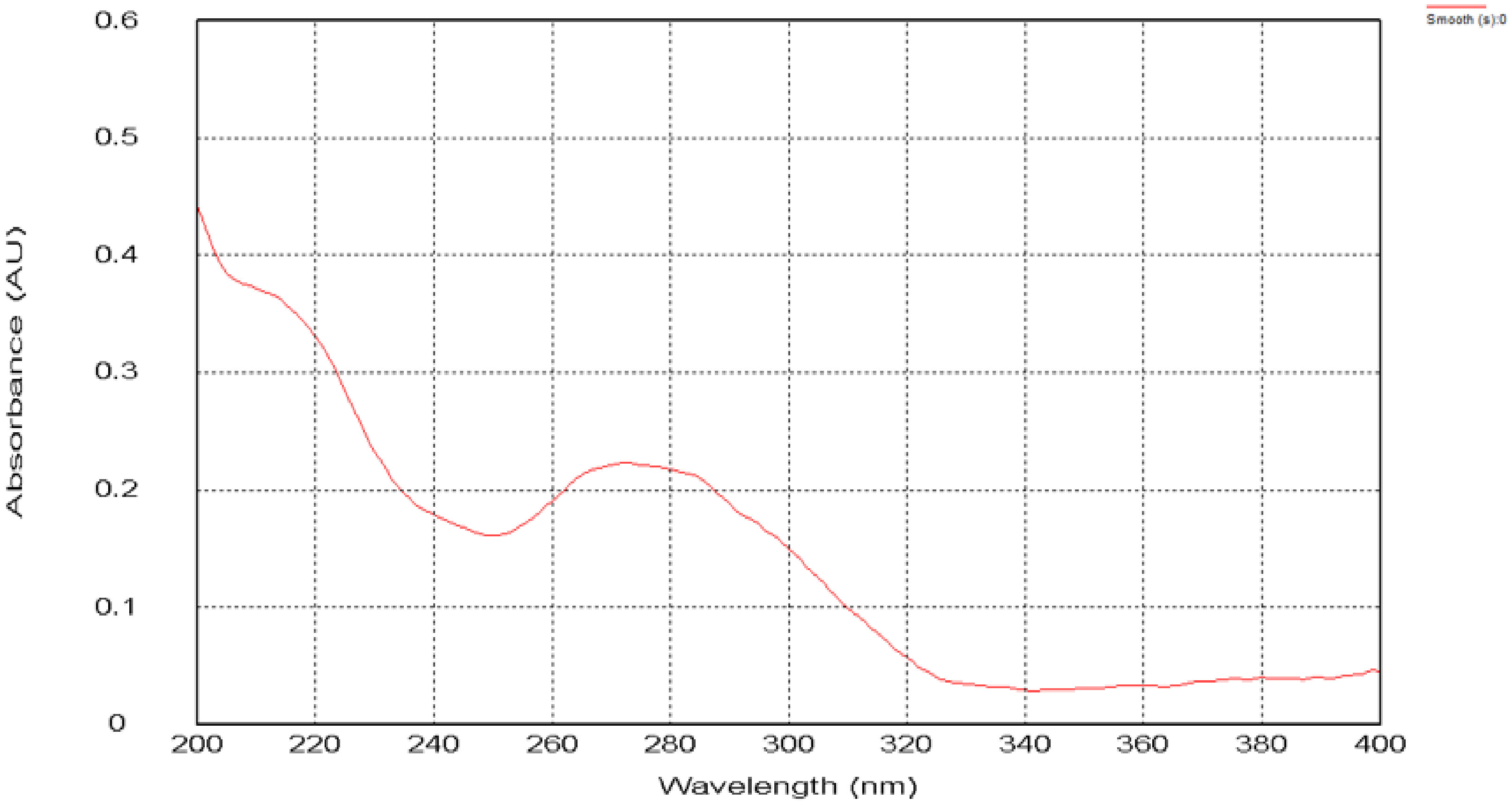

Figure S9. The UV spectrum of compound **2** in MeCN.

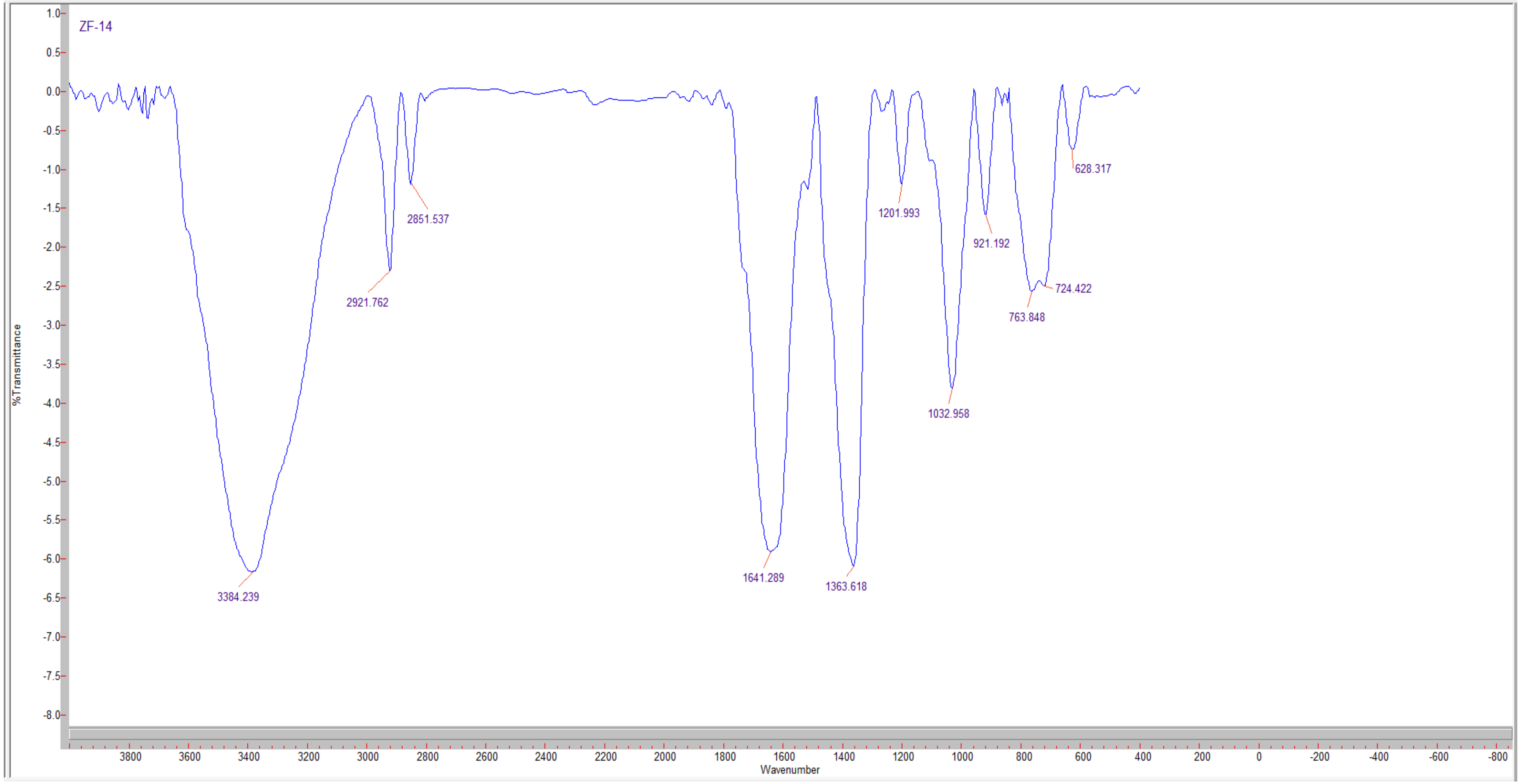

Figure S10. The IR spectrum of compound **2**.

Single Mass Analysis
Tolerance = 1.0 mDa / DBE: min = -1.5, max = 50.0
Element prediction: Off
Number of isotope peaks used for i-FIT = 3
Monoisotopic Mass, Even Electron Ions
329 formula(e) evaluated with 1 results within limits (up to 50 best isotopic matches for each mass)
Elements Used:

Mass	Calc. Mass	mDa	PPM	DBE	Formula	i-FIT	i-FIT Norm	Fit Conf %	C	H	O	Na
587.2061	587.2070	-0.9	-1.5	22.5	C37 H31 O7	472.7	n/a	n/a	37	31	7	

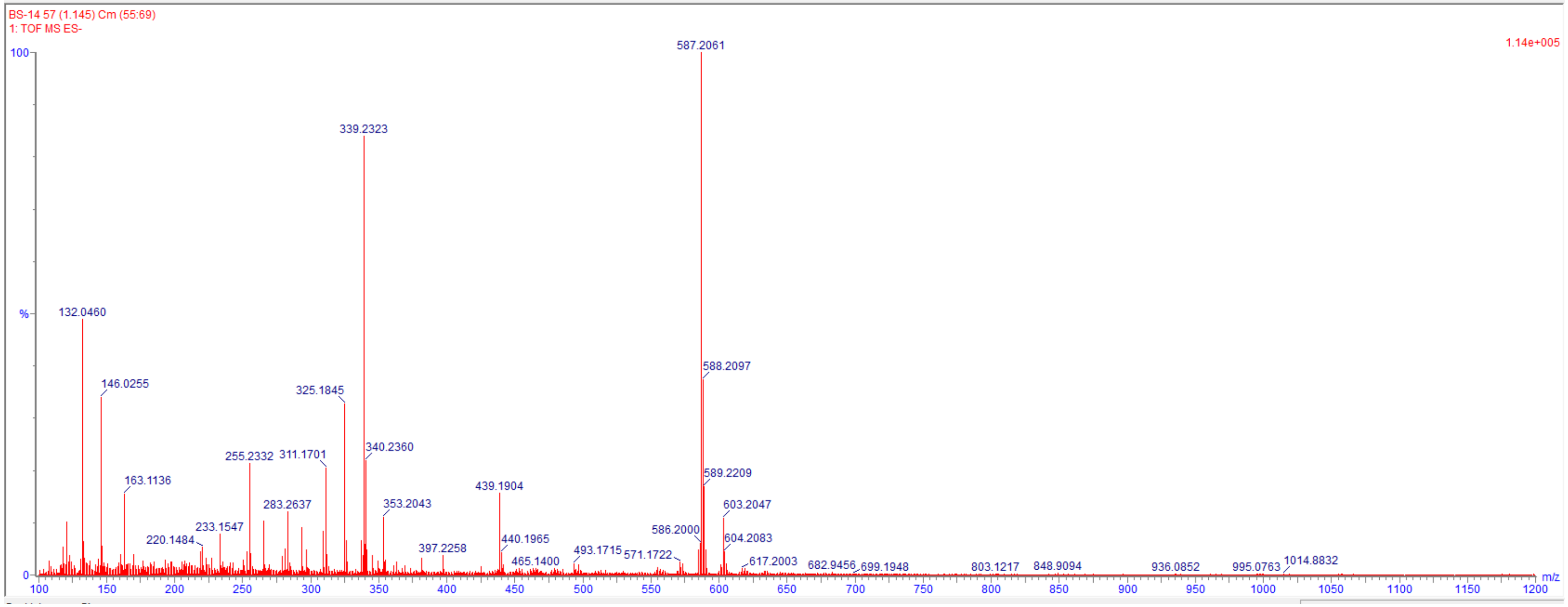

Figure S11. The (–)-HR-ESI-MS spectroscopic data of compound **2**.

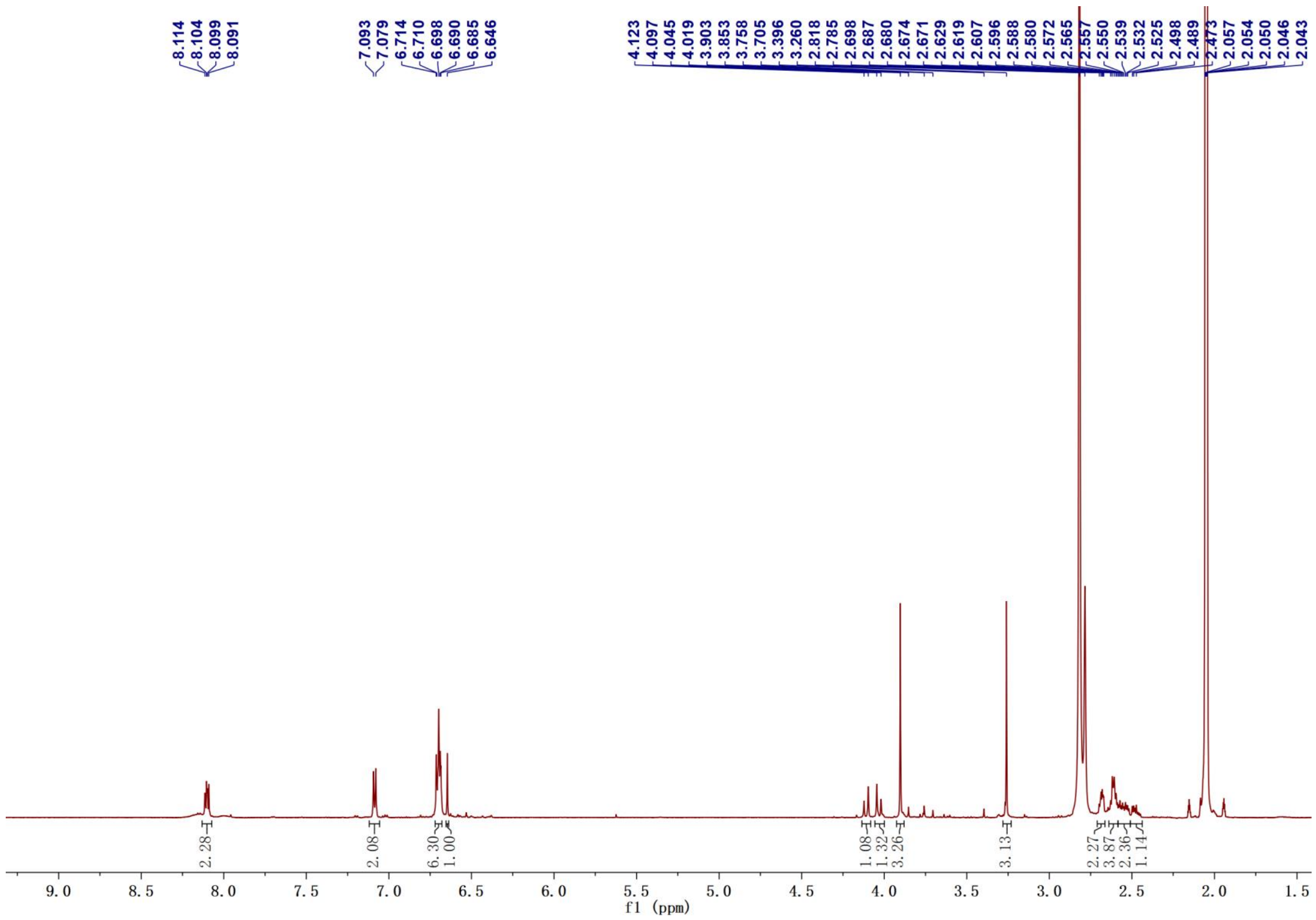

Figure S12. The ^{1}H NMR spectrum of compound **2** in acetone-d_6.

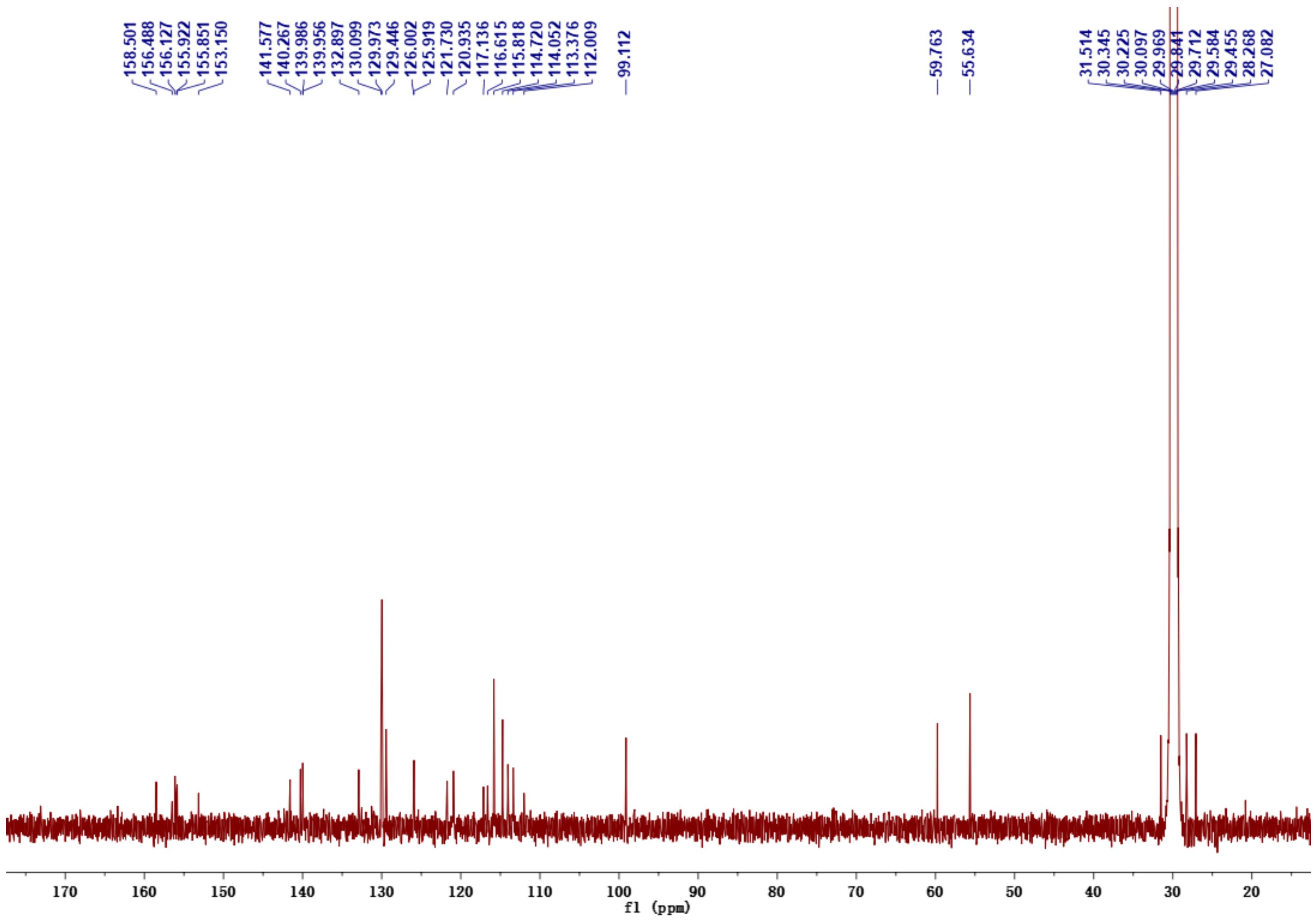

Figure S13. The ^{13}C NMR spectrum of compound **2** in acetone-d_6.

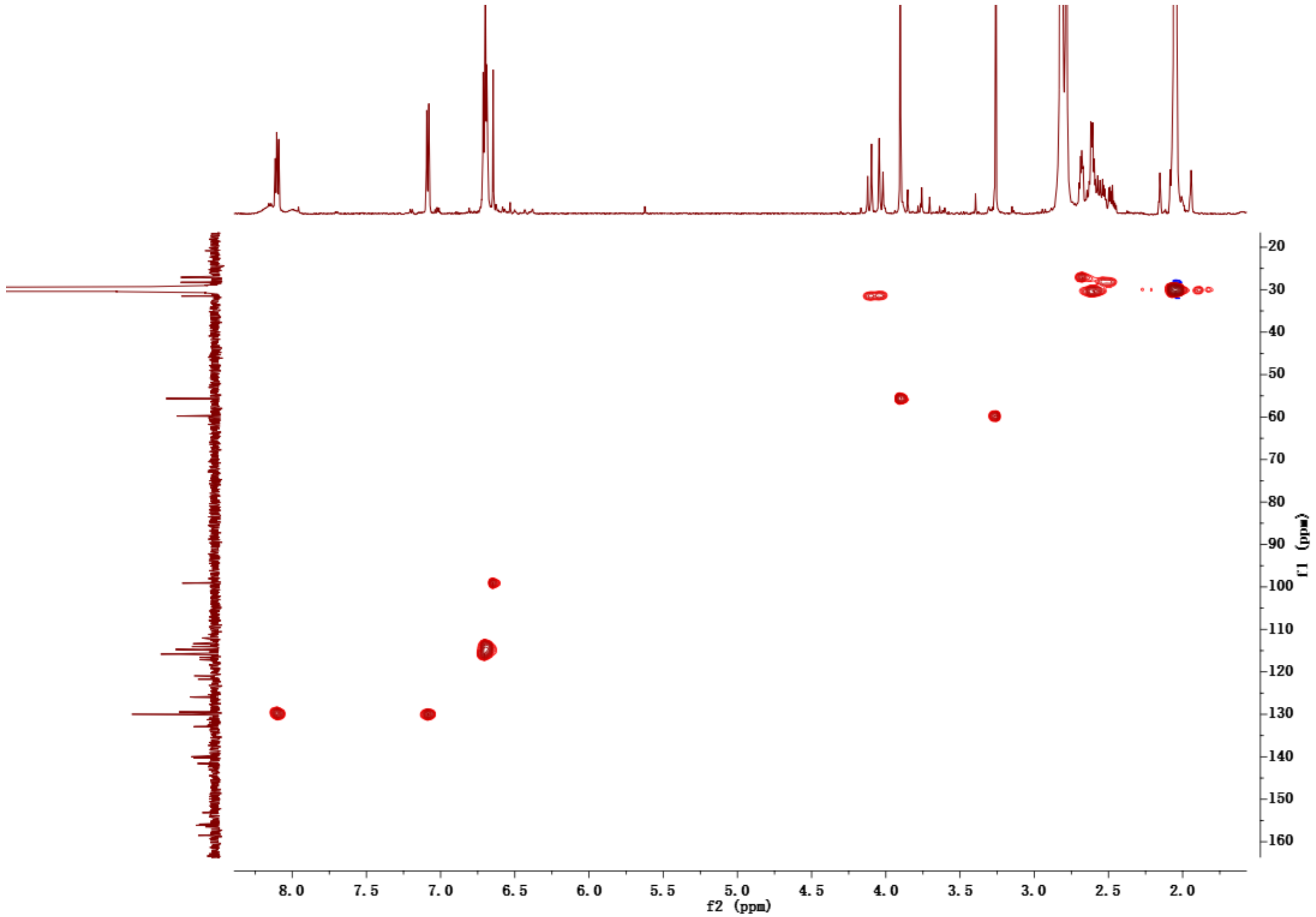

Figure S14. The HSQC spectrum of compound **2** in acetone-d_6.

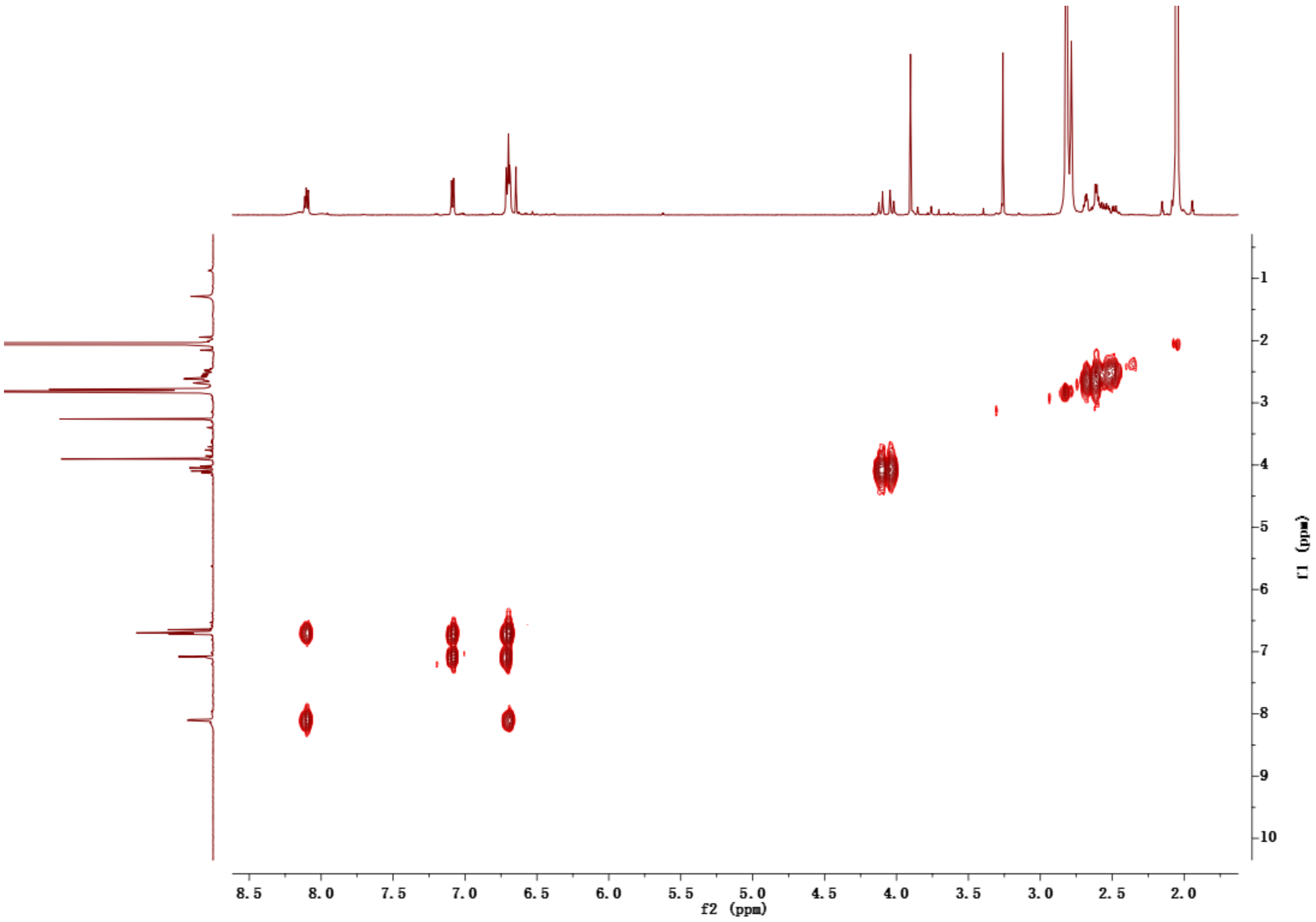

Figure S15. The ^{1}H-^{1}H COSY spectrum of compound **2** in acetone-d_6.

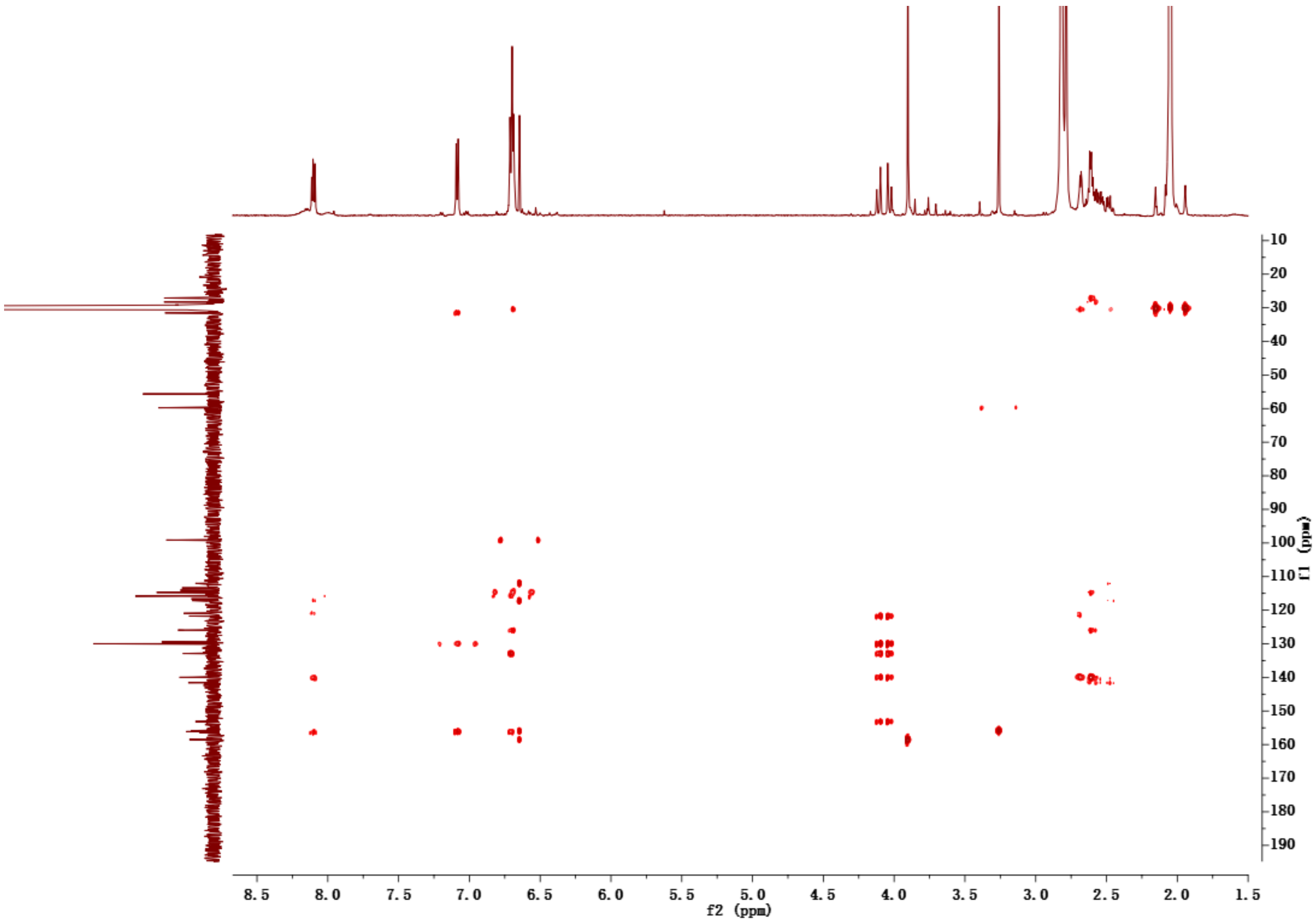

Figure S16. The HMBC spectrum of compound **2** in acetone-d_6.

Table S1. The original western blots in three repetitions for Figure 6 in the paper.

	n_1	n_2	n_3
p-AKT (Ser473)	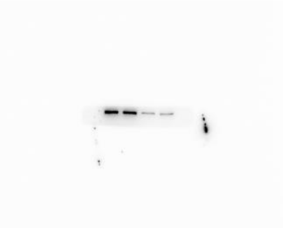		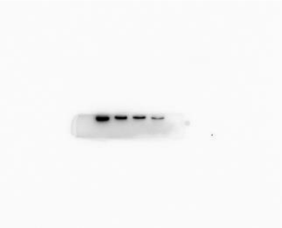
AKT		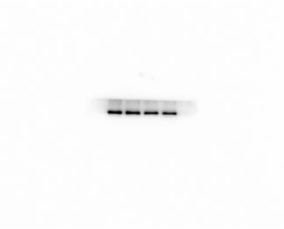	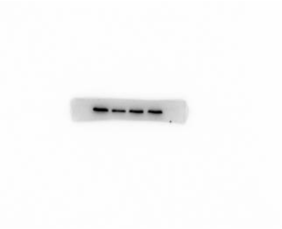
p-MEK1/2 (Ser217/221)	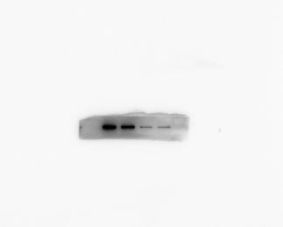	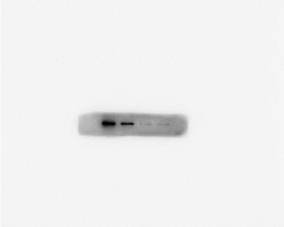	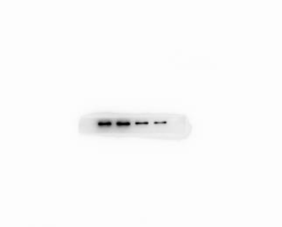

MEK1/2	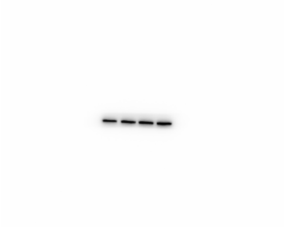	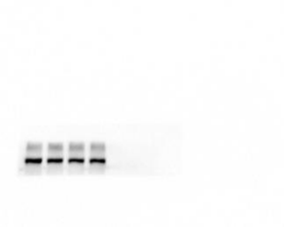	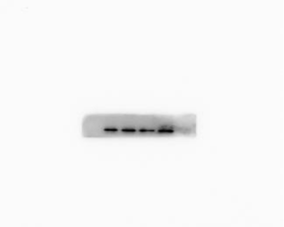
p-ERK1/2 (Thr202/Tyr204)	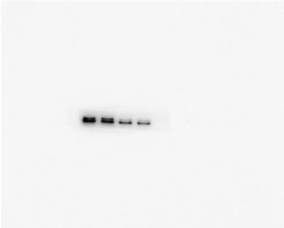	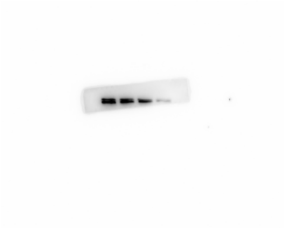	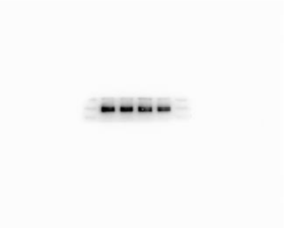
ERK1/2	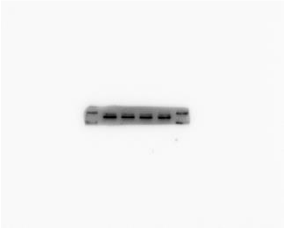	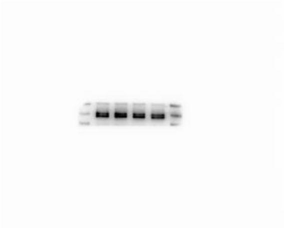	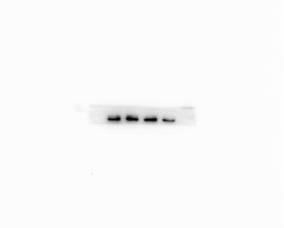
β-actin	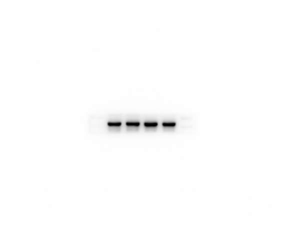	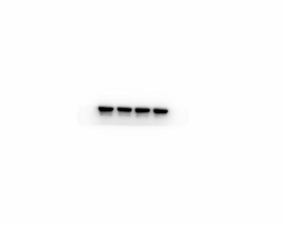	

Table S2. The original western blots in three repetitions for Figure 7 in the paper.

	n_1	n_2	n_3
Bcl-2	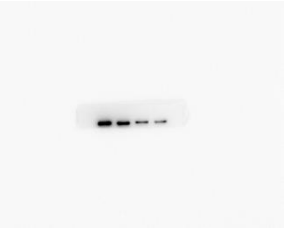		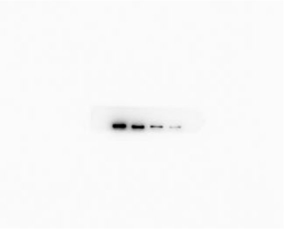
Bax		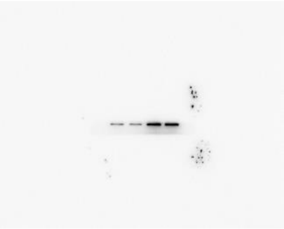	
β-actin		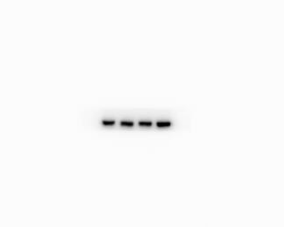	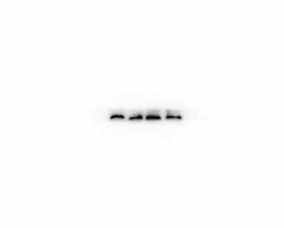
